# A Clade-Specific *Arabidopsis* Gene Connects Primary Metabolism and Senescence

**DOI:** 10.3389/fpls.2016.00983

**Published:** 2016-07-12

**Authors:** Dallas C. Jones, Wenguang Zheng, Sheng Huang, Chuanlong Du, Xuefeng Zhao, Ragothaman M. Yennamalli, Taner Z. Sen, Dan Nettleton, Eve S. Wurtele, Ling Li

**Affiliations:** ^1^Department of Genetics, Development and Cell Biology, Iowa State University, AmesIA, USA; ^2^Department of Statistics, Iowa State University, AmesIA, USA; ^3^Laurence H. Baker Center for Bioinformatics and Biological Statistics, Iowa State University, AmesIA, USA; ^4^Corn Insects and Crop Genetics Research Unit, United States Department of Agriculture-Agriculture Research Service, AmesIA, USA; ^5^Center for Metabolic Biology, Iowa State University, AmesIA, USA

**Keywords:** *SAQR*, stress, senescence, *Arabidopsis*, *QQS*, starch, carbon allocation, AT1G64360

## Abstract

Nearly immobile, plants have evolved new components to be able to respond to changing environments. One example is *Qua Quine Starch* (*QQS*, AT3G30720), an *Arabidopsis thaliana*-specific orphan gene that integrates primary metabolism with adaptation to environment changes. *SAQR (Senescence-Associated and QQS-Related*, AT1G64360), is unique to a clade within the family *Brassicaceae*; as such, the gene may have arisen about 20 million years ago. *SAQR* is up-regulated in *QQS* RNAi mutant and in the *apx1* mutant under light-induced oxidative stress. SAQR plays a role in carbon allocation: overexpression lines of *SAQR* have significantly decreased starch content; conversely, in a *saqr* T-DNA knockout (KO) line, starch accumulation is increased. Meta-analysis of public microarray data indicates that *SAQR* expression is correlated with expression of a subset of genes involved in senescence, defense, and stress responses. *SAQR* promoter::GUS expression analysis reveals that *SAQR* expression increases after leaf expansion and photosynthetic capacity have peaked, just prior to visible natural senescence. *SAQR* is expressed predominantly within leaf and cotyledon vasculature, increasing in intensity as natural senescence continues, and then decreasing prior to death. In contrast, under experimentally induced senescence, *SAQR* expression increases in vasculature of cotyledons but not in true leaves. In *SAQR* KO line, the transcript level of the dirigent-like disease resistance gene (AT1G22900) is increased, while that of the Early Light Induced Protein 1 gene (*ELIP1*, AT3G22840) is decreased. Taken together, these data indicate that *SAQR* may function in the *QQS* network, playing a role in integration of primary metabolism with adaptation to internal and environmental changes, specifically those that affect the process of senescence.

## Introduction

Due to their sessile lifestyle, plants have developed various mechanisms to modulate their internal processes and responses to external stresses by mechanisms including metabolism and senescence. Plants are constantly modifying existing genes and evolving new genes from non-genic sequence; these are thought to enable adaptation to exposure to changing environmental conditions (Neme and Tautz, 2013; Arendsee et al., 2014). Many of the ∼13% of genes in the *Arabidopsis* genome (Lamesch et al., 2012) that encode proteins with no assigned functional motifs and completely unknown functions are relatively new species-specific (orphan) or lineage-specific genes (Gollery et al., 2006; Neme and Tautz, 2013; Arendsee et al., 2014). In recent years, the *Arabidopsis thaliana*-specific orphan gene *Qua Quine Starch (QQS*, AT3G30720) has been revealed as a component of a signaling network that controls metabolic responses to internal and environmental stresses (Li et al., 2009, 2015b; Arendsee et al., 2014; Li and Wurtele, 2015). Several highly lineage-specific genes including Constitutive Expresser of PR Genes 5 (*CPR5*; Jing et al., 2007) and others (Horan et al., 2008; Mentzen and Wurtele, 2008; Luhua et al., 2013; Arendsee et al., 2014) have been shown to be important in enabling an organism to survive under biotic and abiotic stresses.

The *QQS* gene of *A. thaliana* modulates carbon and nitrogen allocation (Li et al., 2009, 2015b; Seo et al., 2011; Arendsee et al., 2014; Li and Wurtele, 2015) via interacting with the evolutionarily conserved transcription factor, nuclear factor subunit C4 (NF-YC4; Li et al., 2015b). Reducing *QQS* expression in *A. thaliana* results in a 15–30% increase in leaf starch content and a 3–7% decrease in protein (Li et al., 2009; Li and Wurtele, 2015), whereas, *QQS* overexpression (OE) decreases starch by as much as 23% and increases protein by 3% (Li and Wurtele, 2015). Neither mutation confers a noticeable effect on plant morphology or development. In addition, *QQS* expression responds actively to abiotic and biotic stress conditions, its transcript level is altered dramatically under those conditions, indicating *QQS* may integrate *A. thaliana* metabolism with responses to stress (Li et al., 2009; Seo et al., 2011; Arendsee et al., 2014; Li and Wurtele, 2015).

Several genes of unknown function have altered transcript abundance in *QQS* RNAi knockdown mutant in *A. thaliana* ecotype Col-0 (Li et al., 2009) in a microarray experiment using Affymetrix ATH1 arrays. One such gene, AT1G64360 (we name it *SAQR*) is up-regulated in *QQS* RNAi lines. This gene is also one of the 119 genes of unknown functions that are up-regulated twofold or more in the ascorbate peroxidase knockout (KO) mutant *apx1*; the *apx1* mutant shows an increased susceptibility to light-induced oxidative stress (Davletova et al., 2005). APX1 (AT1G07890), a cytosolic hydrogen peroxide scavenger, was found to be essential for chloroplastic protection from reactive oxygen species damage and sufficient for this protection in the absence of stromal/mitochondrial APX relatives (Davletova et al., 2005). *SAQR* expression in leaf is up-regulated about twofold under oxidative stress, ABA (abscisic acid) treatment and heat stress conditions, but does not change much under osmotic stress, salt stress, and cold stress conditions (Luhua et al., 2008). *SAQR* expression in root is up-regulated about sixfold to osmotic stress, and responds to oxidative stress, salt stress, cold stress, ABA treatment, and heat stress (up-regulated about twofold). But *SAQR-OE* lines do not show significantly increased tolerance to oxidative stress (Luhua et al., 2008). *SAQR-OE* lines flower earlier under short day (SD) conditions compared to controls (Luhua et al., 2008).

The altered expression of *SAQR* under light-induced oxidative stress, its relatively recent origin (with homologs in only five other genomes), and the potential relationship between *SAQR* and *QQS* motivated this study. Our working hypothesis was that *SAQR* plays a role in the QQS network. Here, we use a combination of genomic, bioinformatic, transcriptomic, and molecular approaches to further characterize the *SAQR* gene in relation to senescence, metabolism, and stress responses in the plant.

## Materials and Methods

### Plant Materials, Growth and Transformation

Constructs of SAQR promoter::GFP/GUS (promoter region includes 715 bp upstream of *SAQR* start codon) and 35S::SAQR coding sequence (CDS) were generated using the Gateway system (Life Technologies) as previously described (Li et al., 2007; Li and Wurtele, 2015). The primers used were: 5′-AAAGCTTGATGGAGAAGAAAAGGT-3′ and 5′-TGTTTCACCTGCTAAGTGTCTTT-3′ for SAQR promoter::GFP/GUS, 5′-ATGTCGTTTAGAAAAGTAGAGAAGAA-3′ and 5′-TTAGTAATTAGGGAAGTGTTTGCG-3′ for 35S::SAQR CDS. SAQR T-DNA KO (SALK_052233C, *saqr*) germplasm was ordered from the *Arabidopsis* Biological Resource Center (ABRC^1^).

Transgenic *A. thaliana* plants (ecotype Columbia-0, Col-0) were generated using the floral dipping method (Clough and Bent, 1998) and selected as previously described (Li et al., 2007). Plants were grown in Sun Gro Sunshine LC1 soil mix in pots in flats in a greenhouse room at 22°C under constant fluorescent light, of approximately 130 μmol m^-2^ s^-1^ for most experiments. Similar conditions but an 8 h light/16 h dark cycle was used for the SD flowering experiment. For starch content experiments, plants were germinated on 0.5X Murashige and Skoog medium plates supplemented with 1% sucrose, transferred to pots with soil and grown in a growth chamber at 22°C under fluorescent light of approximately 130 μmol m^-2^ s^-1^ using a long day (LD) conditions of 16 h light/8 h dark.

### 5′ and 3′ RACE

Rapid amplification of cDNA ends (RACE) experiments were performed to define the 5′ and 3′ UTRs (untranslated region) of the *SAQR* gene as previously described (Li et al., 2009). The primers used were: 5′-CGACTGGAGCACGAGGACACTGA-3′ and 5′-GAAACGAAGACATGCAGGCTC-3′ for the 5′ UTR product, 5′-ACCAAGGCAATACATTTTACCTAA-3′ and 5′-GCTGTCAACGATACGCTACGTAACG-3′ for the 3′ UTR product.

### Bioinformatics Analysis

MetaOmGraph was used to analyze the transcriptomic expression pattern of *SAQR* using the normalized experimental data and metadata (metadata includes gene, experiment and sample annotations) from 71 experiments comprising 956 Affymetrix ATH1 microarray arrays [dataset “At956-2008” (Li et al., 2007, 2009; Mentzen and Wurtele, 2008)]. MetaOmGraph is available online^2^

*Cis*-acting motifs present within the *SAQR* promoter region upstream of the transcription start site were analyzed using Athena (O’Connor et al., 2005), Plant Care^3^, and the Plant Promoter Database (Yamamoto and Obokata, 2008).

### Histochemistry

Twelve independent *SAQR* promoter-GUS lines were screened by GUS staining. At least five transgenic plants from each of at least three representative independent *SAQR* promoter-GUS lines were harvested at separate stages of development and from the induction experiments. The plants were stained according to a protocol as previously described (Li et al., 2007). Similarly appearing seedlings were selected, one unstained was photographed and five were processed to be stained. Staining patterns were observed using a Zeiss Axio Zoom microscope at the Iowa State Microscopy and NanoImaging Facility (Ames, IA, USA).

### Molecular Methods

Starch content was analyzed qualitatively by staining plants just before flowering with I_2_/KI as previously described (Li et al., 2009), and quantified using an amyloglucosidase/α-amylase and GOPOD (Megazyme) protocol (Li et al., 2009). Experiments were performed with two independent T_2_ *SAQR-OE* lines, SALK_052233C (*saqr*), and wild type (WT) plants, with five plants per genotype per replicate, and three replicates per genotype. This experiment was repeated twice.

Plants/leaves were treated by one of three different dark-stress protocols to induce senescence. For whole seedlings, plants were grown for 1 week, and then covered with aluminum foil for 5 days and exposed to light for 4 days (WPD); controls were kept under constant light under the same conditions (Weaver and Amasino, 2001). For attached leaves, fully expanded true leaves attached to 12-DAI (days after imbibition) plants were carefully covered with aluminum foil for 3 days (DIS; van der Graaff et al., 2006). For detached leaves, fully expanded true leaves were detached from 12-DAI plants and floated on water in a Petri dish covered with aluminum foil for 3 days (DET; van der Graaff et al., 2006). Leaves in similar positions on untreated plants were used as controls.

For experiments with stress or hormone treatment, seedlings were excised at 12 DAI into water and either untreated or treated with 1 μM kinetin (cytokinin; Coenen and Lomax, 1998), 500 μM hydrogen peroxide (oxidative stress; Luhua et al., 2008), 10 μM methyl jasmonate (JA; Staswick et al., 1992), or 50 μM 1-aminocyclopropane-1-carboxylic acid (ACC; ethylene; Beaudoin et al., 2000) for 4 days. For salt treatments, flats of 12-DAI plants in pots were allowed to dry till they were slightly light in weight and then watered with either filtered water or water containing 200 mM NaCl (Wu et al., 1996) and observed after 4 days. Drought stress was simulated by allowing seedlings to go unwatered for 15 days until wilted.

### RNA-Seq

The SAQR-KO (*saqr*) line SALK_052233C and WT plants were grown and harvested at 20 DAI, at the end of the light cycle under LD conditions as previously described (Li et al., 2015b). Independent randomizations for plant growth and harvest were used for each of two biological replicates. The RNAs were extracted and purified as previously described (Li et al., 2015b). The 200-bp short-insert library and the transcriptome sequencing were conducted at BGI Americas^4^ as described before (Li et al., 2015b). The cleaned reads were aligned, mapped reads were counted, and genes were tested for differential expression to compare *saqr* and WT. *P*-values and *Q*-values were generated as previously described (Li et al., 2015b). The three genes with *P*-values less than 0.00001 were considered to be differentially expressed, which led to false discovery rate control at approximation 13% in this experiment. RNA-Seq data have been deposited in the NCBI Sequence Read Archive^5^, accession number: SRP072428.

## Results

### Evolutionary and Structural Characterization of SAQR

Senescence-Associated and QQS-Related is a single copy gene that encodes an 85 amino acid protein. Five other sequenced genomes have *SAQR* homologs: *A. lyrata*, *A. halleri*, *Capsella rubella*, *C. grandiflora*, and *Boechera stricta* (**Supplementary Figure S1**). Each of these species is in the *Brassicaceae* family within a monophyletic clade (Mitchell-Olds et al., 2005; Windsor et al., 2006). No SAQR homologs were detected in other eukaryotes or prokaryotes, including two other sequenced members of *Brassicaceae* (Yang et al., 2013): *Brassica rapa* and *Eutrema salsugineum*.

**FIGURE 1:**
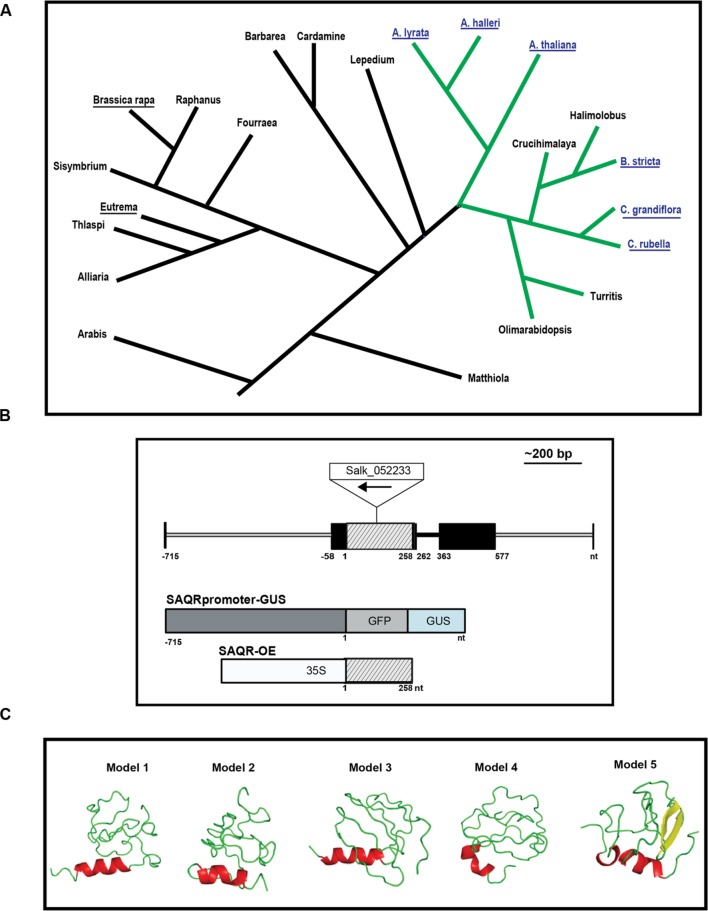
**Senescence-Associated and QQS-Related (*SAQR*) gene and predicted protein.** **(A)** *SAQR* has homologs in a monophyletic group within family *Brassicaceae*. Blast searches of the NCBI database show three genera contain a *SAQR* homolog: *Arabidopsis*, *Capsella*, and *Boechera*. Blue font, genomes containing an *SAQR* homolog. Underlined names, species with sequenced genomes. Green lines, the monophyletic group containing SAQR. Simplified tree structure adapted from Koch and Kiefer (2005), Clauss and Koch (2006), Schranz et al. (2006), Windsor et al. (2006). **(B)** *SAQR* gene model, as determined by 5′ and 3′ RACE. Black boxes, 5′ and 3′ UTR; lined gray box, coding region; black line, intron; gray line, non-transcribed region. Constructs used to make the *SAQR* promoter-GUS and *SAQR-OE* lines are pictured in relation to the gene model. The BAR, 35S, and GUS/GFP reporter are not to scale. Nucleotide positions numbered in relation to the ATG start codon of *SAQR*. **(C)** Structural models of the SAQR protein predicted using I-TASSER. Helices are colored red; sheets, yellow; loops, green. Image made using PyMol (DeLano and Bromberg, 2002).

The six species that possess a *SAQR* homolog belong to a lineage of organisms that separated from the lineage containing the *Brassica* and *Eutrema* genera about 20 million years ago (MYA; Clauss and Koch, 2006; Domazet-Lošo et al., 2007; Arendsee et al., 2014). The monophyletic group that contains these six species also includes the genera *Turritis*, *Olimarabidopsis*, *Halimolobus*, and *Crucihimalaya* (**Figure 1A**). It is possible that these genera also contain a *SAQR* homolog, but full genomes of members of these genera were not publicly available as of June 12, 2016.

To experimentally confirm the *SAQR* mRNA sequence, RACE and RT-PCR experiments were conducted using RNA from *A. thaliana* Col-0 rosette leaves at the beginning of flowering. The enriched mRNA covered the entirety of the *SAQR* CDS (**Figure 1B**, **Supplementary Figure S2**). The 5′ UTR is identical to that of the TAIR10-predicted model, including 58-bp nucleotides upstream of the reported translational start site. The 3′ UTR extends 577 bp downstream of the stop codon, which is 29 bp shorter than the TAIR10-predicted gene model. The *SAQR* homologs in *Arabidopsis* species and *Boechera stricta* have a similar gene structure: they are generally conserved in the 5′ UTR, CDS, and 3′ UTR regions, and the encoded proteins are similar in length and sequence. In contrast, the translation start site for the *Capsella* variant is predicted to start from an ATG farther upstream in the 5′ UTR than the start codon for the other four species, thus the *Capsella SAQR*-like protein has an additional 38 aa in the N terminal. All *SAQR* homologs identified have a single intron that follows immediately after the stop codon (**Figure 1B**).

The *cis*-acting motifs in the *SAQR* promoter region upstream of the transcription start site (-715 to -58 bp; **Figure 1B**) were analyzed. The analyses indicate that this 658-bp promoter region contains 27 *cis*-acting motifs (**Supplementary Table S1**). These include two binding sites for AGAMOUS-LIKE 15 (AGL15). AGL15 is a nuclear protein that delays flowering and senescence when overexpressed (Fang and Fernandez, 2002). The early flowering phenotype of *SAQR-OE* mutants (Luhua et al., 2008) may be associated with the presence of this motif. The *SAQR* promoter also has 10 light-responsive/circadian-associated regions, and multiple stress-related motifs: a HEAT-SHOCK ELEMENT (HSE) *cis*-motif that can induce genes in response to heat shock, oxidative stress, and other stresses (Storozhenko et al., 1998); binding sites for DEHYDRATION-RESPONSIVE ELEMENT BINDING (DREB) proteins; an ABA signaling motif; and a salicylate response motif.

The SAQR protein has no conserved domains. Secondary structure predictions using I-Tasser (Roy et al., 2010) indicate that SAQR may be composed of 10% α-helix and up to 10% of β-strands, while the major part of the protein (78–91%) is predicted in the loop region (**Supplementary Table S2**); a single helical region is predicted (**Figure 1C**). Analysis of the SAQR protein sequence using MetaDisorderMD2 (Kozlowski and Bujnicki, 2012) indicates that it has a largely disordered structure within two regions between amino acids 1–29 and 71–85, a somewhat more ordered section within amino acids 43–57, and a global disorder tendency of 0.642 (**Supplementary Figure S3A**). “Disordered” denotes proteins lacking a fixed tertiary structure. Interestingly, disorder does not appear to be evolutionarily stable under random processes, and must be specifically selected for (Schaefer et al., 2010); one of the most highly conserved proteins in the plant kingdom, the LATE EMBRYOGENESIS ABUNDANT (LEA) protein, EMB1 (Wurtele et al., 1993) is also one of the most disordered (Eom et al., 1996). The term “LEA” is now broadly used to referred to genes in any of the multiple families of genes that are abundant during embryo desiccation, and LEAs, including EMB1, are thought to change to an ordered conformation under desiccation or cryodamaging conditions, and act to stabilize cellular structures and molecules (Eom et al., 1996; Reyes et al., 2005; Battaglia et al., 2008; Olvera-Carrillo et al., 2011).

The predicted physical characteristics of the SAQR protein (thought not its aa sequence) are reminiscent of a class of LEA-like stress proteins called hydrophilins (López-Martínez et al., 2012): a relatively small size (SAQR is 85 aa), a glycine content greater than 6% (SAQR is 9.4%), a high hydrophilicity index (**Supplementary Figure S3B**), and a predicted structure dominated by large disordered regions and coils. Some LEAs, including several members of sub-groups of the hydrophilins, have been experimentally shown to confer resistance to osmotic stress and other abiotic stressors (Shinozaki et al., 2003; Battaglia et al., 2008). A senescence-associated LEA, *SAG21* (SENESCENCE ASSOCIATED GENE 21; *LEA5*), is localized in mitochondria and up-regulated under biotic and abiotic stresses; *SAG21* antisense plants flower earlier under LD conditions (Salleh et al., 2012).

The five stress response motifs in the promoter region of *SAQR* and the hydrophilin-like physical characteristics of SAQR protein, implicate the *SAQR* gene may play a potential role in stress response. This finding led us to evaluate the expression patterns of the *SAQR* gene in *Arabidopsis* under conditions of developmental and environmental stresses.

### *SAQR* Transcript Accumulation Profile is Influenced by Senescence and Stress

**FIGURE 2:**
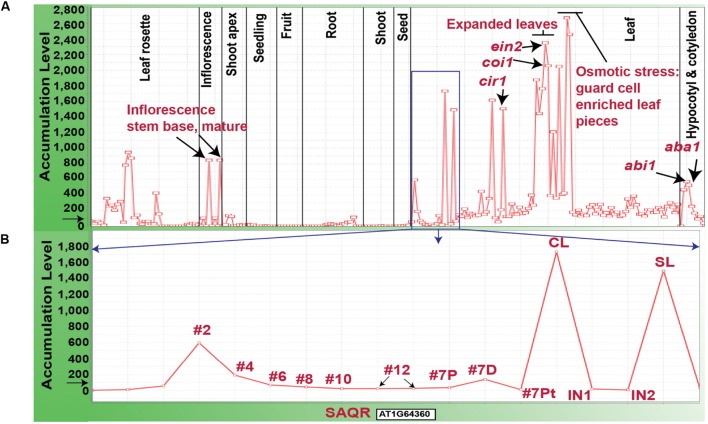
**Accumulation of *SAQR* (AT1G64360) mRNA.** **(A)** *SAQR* expression across 956 developmental stages, environmental treatments and genetic mutations. **(B)** *SAQR* expression in leaves at different development stages. **(B)** Represents data within the blue square in **(A)**. Samples were taken from leaf number #2-#12 in 17-DAI plants (the lower numbered leaves are older; Schmid et al., 2005). Leaf #7 of 17-DAI plants is divided into: 7P, proximal half, 7D distal half, and 7Pt, petiole; the distal part of the leaf contains the oldest tissue. CL, cauline leaf of 21-DAI plants; SL, senescent rosette leaf of 21-DAI plants; IN1, internode 1; IN2, internode 2. Each point on the X-axis represents *SAQR* expression in a given tissue sample. The Y-axis represents the normalized expression level for the *SAQR* gene, mean expression level for all genes across the chip is normalized to 100, as indicated by the black arrow. mRNA transcriptome profiling dataset “At956-2008” is visualized using MetaOmGraph software (http://www.metnetdb.org).

Our microarray experiment revealed that the *SAQR* transcript accumulates to >2-fold greater levels in *QQS* RNAi mutant compared to WT control plants, which indicates that *SAQR* transcript accumulation is negatively influenced by *QQS*. We evaluated global *SAQR* expression (**Figure 2A**) using MetaOmGraph^enumfont 2^ and a large public microarray dataset “At956-2008” (Li et al., 2007, 2009; Mentzen and Wurtele, 2008). Under standard growth conditions in WT plants, as shown in **Figure 2A**, *SAQR* expression is highest in fully expanded leaves, at the base of the mature inflorescence, in senescing leaves, and cauline leaves. Expression is moderate within the hypocotyl and the plant rosette prior to flowering. *SAQR* accumulation is below detection limits in the roots, developing fruits, and very young seedlings and seeds.

In addition, *SAQR* expression is increased after plant exposure to drought stress, and under high-osmotic conditions in leaf pieces enriched with guard cells. Expression of *SAQR* is also increased in specific mutants of hormone metabolism or signaling. These mutants include: *abi1*, ABA insensitive (Wu et al., 2003); *aba1*, ABA deficient (Koornneef et al., 1982; Niyogi et al., 1998); *ein2*, ethylene insensitive (Oh et al., 1997); *coi1*, JA insensitive mutant (He et al., 2002); *cir1*, which has altered sensitivity to ethylene, JA, and salicylate (Murray et al., 2002); and *myb29*, MYB29 promotes glucose-induced biosynthesis of aliphatic glucosinolates (Miao et al., 2013). *SAQR* expression is down-regulated by 53-fold in a *mute* background; the *MUTE* gene is required for stomatal development (de Marcos et al., 2015).

Individual leaves undergo mitotic growth, expansion, senescence, and death (Lim et al., 2007). Unlike for many other species, these processes are minimally influenced by the reproductive status of the *Arabidopsis* plant (Noodén and Penney, 2001). Therefore, during vegetative growth and reproduction, individual rosette leaves of an *Arabidopsis* plant are at varying stages of the maturity/senescence program. *SAQR* expression is lower in younger leaves and higher in the oldest leaves of plants of the same age (**Figure 2B**; microarray data from Schmid et al., 2005). *SAQR* is also more highly expressed in the distal (older) section of a moderately mature leaf, compared to the petiole or proximal section of that leaf.

This increased accumulation of *SAQR* transcript in leaves that are transitioning from expansion to senescence, under some stress conditions, and in several mutants of genes of stress hormone synthesis or signaling, further supports the relationship between *SAQR* and senescence/stress.

### Processes Overrepresented among Genes Co-expressed with *SAQR*

To further investigate the potential function of *SAQR*, we identified the genes that are highly co-expressed with *SAQR* and then evaluated the overrepresentation of regulons and pathways among these genes. To do this, we used the Spearman’s correlation function in MetaOmGraph. We chose Spearman’s correlation to avoid the major shortcoming of Pearson’s correlation—sensitivity to outliers (Mukaka, 2012). This analysis indicates that 133 genes had a *positive* correlation coefficient of >0.7 with *SAQR* across multiple environmental, genetic and developmental conditions.

*Arabidopsis* genes have been globally classified into regulons by pairwise co-expression analysis of the “At956-2008” microarray dataset followed by Markov Chain Clustering (MCL) of the resultant co-expression matrix (Mentzen and Wurtele, 2008). Regulons in eukaryotes can be defined as groups of genes that are co-expressed across multiple environmental, developmental and genetic conditions; genes in a regulon are predicted to play roles in a particular process, such as systemic acquired resistance, oxidative respiration, leucine catabolism, or sperm cell differentiation (Mentzen and Wurtele, 2008; Mentzen et al., 2008; Borg et al., 2011). Distinct from the concept of *pathways*, in which the genes have a known biochemical function and a known relationship to one another, *regulons* are derived from a computational clustering of co-expressed genes; these genes could code for, *e.g.*, regulatory, catalytic, structural, or signaling proteins or non-coding RNAs. In the analysis of Mentzen and Wurtele (2008), regulons were numbered by size, and a predominant function/process was assigned to each regulon based on overrepresentation analysis of the annotations for the genes with known function in that regulon. The genes within a regulon with no prior known function can be considered potential candidates to play a role in the function/process assigned to that regulon. For example, the *FAP1-3* genes were members of a regulon assigned as fatty acid biosynthesis, based on the preponderance of genes in that regulon being enzymes of fatty acid biosynthesis (Mentzen et al., 2008); this regulon membership led to experimental analysis that identified the *FAP* genes as regulators of fatty acid biosynthesis (Ngaki et al., 2012).

After identifying the genes that are co-expressed with *SAQR*, we checked for overrepresentation of regulons among them (**Table 1** and **Supplementary Table S3**). Twenty-eight of the genes co-expressed with *SAQR* are involved in defense responses; this includes almost 70% of the genes in defense-related Regulon #25. Ten of the *SAQR*-co-expressed genes are in signaling/disease resistance-related Regulon #35 (23% of the genes in that regulon), and 12 in phloem/vascular tissues Regulon #57 (57% of the genes in that regulon). Most other *SAQR*-co-expressed genes are grouped within smaller regulons of unspecified function, or are members of the large photosynthesis regulon (#2). Six *SAQR*-co-expressed genes are not members of any regulon (they comprise < 0.04% of this large gene group).

**Table 1:** Regulons overrepresented among genes with expression patterns positively correlated with that of *SAQR*.

Regulon	Number of genes in regulon positively correlated with *SAQR*	Total number of genes in regulon	% of regulon genes positively correlated with *SAQR*
25 – Defense response	28^∗∗^	69	40
2 – Photosynthesis	24^∗∗^	1135	2
57 – Phloem specific (vasculature tissues – specific)	12^∗∗^	21	57
35 – Kinases, signaling, disease resistance	10^∗∗^	44	23


*The 133 genes with a positive correlation coefficient of >0.7 were classified by gene membership in co-expression regulons (Mentzen and Wurtele, 2008). *P-*values were calculated within R, using the Fisher’s Exact Test. mRNA transcriptome profiling dataset “At956-2008” was used. Overrepresentation ^∗∗^*P*-values < 0.001.*

Using the same “At956-2008” dataset, we identified 134 genes whose expression patterns had a *negative* correlation coefficient (< -0.6) with that of *SAQR*, and determined the regulon membership of this group of genes (**Table 2** and **Supplementary Table S4**). Over one third of the genes that negatively correlate with *SAQR* are members of the mitosis regulon (#4), and two are in the nuclear replication/chromosome organization regulon (#47). These two processes would likely be minimal during senescence or in times of stress.

**Table 2:** Regulons overrepresented among genes with expression patterns negatively correlated with that of *SAQR*.

Regulon name	Number of genes negatively correlated with *SAQR*- in regulon	Total number of genes in regulon	% of regulon genes negatively correlated with *SAQR*
4 – mitosis	51 ^∗∗^	582	9
47 – nuclear, replication, chromosome organization	2 ^∗^	26	8


*The 134 genes with a negative correlation coefficient of < -0.6 were classified by gene membership in co-expression regulons (Mentzen and Wurtele, 2008). *P-*values were calculated within R, using the Fisher’s Exact Test. mRNA transcriptome profiling dataset “At956-2008” was used. Overrepresentation ^∗∗^*P*-values < 0.001 and ^∗^*P* < 0.05.*

In a second approach to develop hypotheses on SAQR function, we identified pathways that are overrepresented among the genes that are correlated with *SAQR* expression across multiple conditions. For this, we used MetNet tools (Sucaet et al., 2012; Li and Wurtele, 2015; Li et al., 2015a) and the “At956-2008” dataset (Li et al., 2007, 2009; Mentzen and Wurtele, 2008). AraCyc^6^, AGRIS^7^, and MetNet^8^ pathways/networks were evaluated; since there are no pathways specifically designated as developmental or stress-response processes such as “mitosis” or “flowering” or “defense against bacteria” in these annotations, the overrepresentation of such processes would not be detected by this approach.

Pathways that are highly overrepresented among the 1,250 genes (a *positive* Spearman correlation coefficient > 0.5 with the *SAQR* transcript; **Table 3**) include pathways involved in the synthesis and signaling of the defensive/stress-related responses: JA signaling; camalexin, traumatine, ornithine, and glucosinolates. Photosynthesis-related pathways (chlorophyll degradation, oxygenic photosynthesis, photosynthesis light reactions, photorespiration, sucrose synthesis, and the Calvin cycle) are also overrepresented. These overrepresented pathways overlap in part with the overrepresented regulons of the genes that are positively co-expressed with *SAQR* in analysis (**Table 1**), in which photosynthesis and defense regulons are well-represented. The pathways overrepresented among the 596 genes negatively correlated with *SAQR* expression (a *negative* Spearman correlation coefficient < -0.5 with the *SAQR* transcript) include glycolysis, gluconeogenesis, auxin degradation, isoleucine degradation, and the mevalonate pathway (**Table 4**).

**Table 3:** Pathways overrepresented among the transcripts positively co-expressed with *SAQR*.

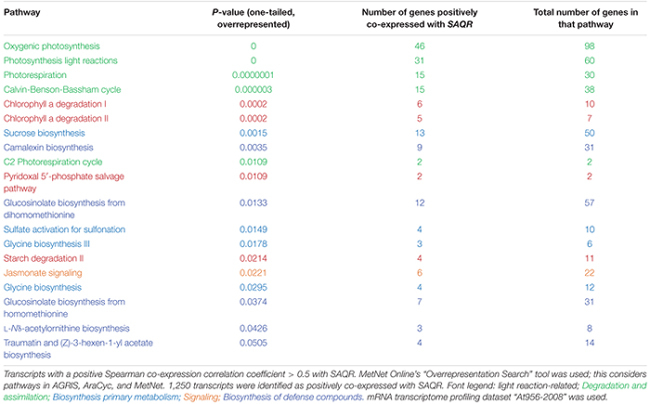

**Table 4:** Pathways overrepresented among the transcripts negatively correlated with *SAQR*.

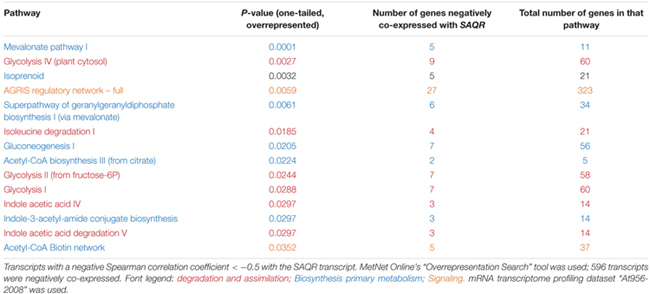

Many of the overrepresented pathways among *SAQR*-co-expressed genes are related to senescence. Decreases in primary metabolic and photosynthetic pathways are tightly linked to senescence (Buchanan-Wollaston et al., 2003; Lim et al., 2007). JA signaling participates in regulating senescence, as well as pathogen stress, and JA application can induce senescence (He et al., 2002; Devoto and Turner, 2003). The JA signaling mutant *coi1*, in which the *SAQR* transcript is increased (**Figure 2A**), shows delayed leaf senescence (Buchanan-Wollaston et al., 2005). The auxin pathway, overrepresented among genes negatively correlated to *SAQR*, may delay senescence (Mueller-Roeber and Balazadeh, 2014). The overrepresentation of mitosis among those genes negatively correlated to *SAQR* (**Table 2**) would be expected of mature or senescing tissue, as cell division is curtailed in the later stages of the life cycle of *Arabidopsis* leaves (Gonzalez et al., 2012).

These findings led us to examine the relationship between senescence and *SAQR*. To do this, we focused on senescence-associated genes (SAGs), defined as genes that are differentially expressed when senescence occurs naturally and/or is induced by darkness (van der Graaff et al., 2006). Some SAGs have a defined function, but notable percentages have no known function. The approximately 2,900 SAGs that are differently regulated during natural senescence but not under induced senescence include genes in the JA, ethylene, and salicylic acid metabolic pathways (van der Graaff et al., 2006; Breeze et al., 2011; Allu et al., 2014) as well as *SAQR* itself. Fifty-three genes – 40% of all of the genes that are positively co-expressed with *SAQR* – are SAGs that are up-regulated under *natural* senescence but not under *induced* senescence (**Supplementary Table S5**). In contrast, very few genes that are negatively co-expressed with *SAQR* are up-regulated under *induced* senescence (**Supplementary Table S6**); also, few *SAQR*-co-expressed genes are down-regulated under conditions of either natural or induced senescence (**Supplementary Tables S5** and **S6**). These findings are consistent with a relationship between *SAQR*, *SAQR*-co-expressed genes and plant natural senescence.

### *SAQR* is Expressed in Vasculature of Maturing and Senescing Leaves and Tissues

**FIGURE 3:**
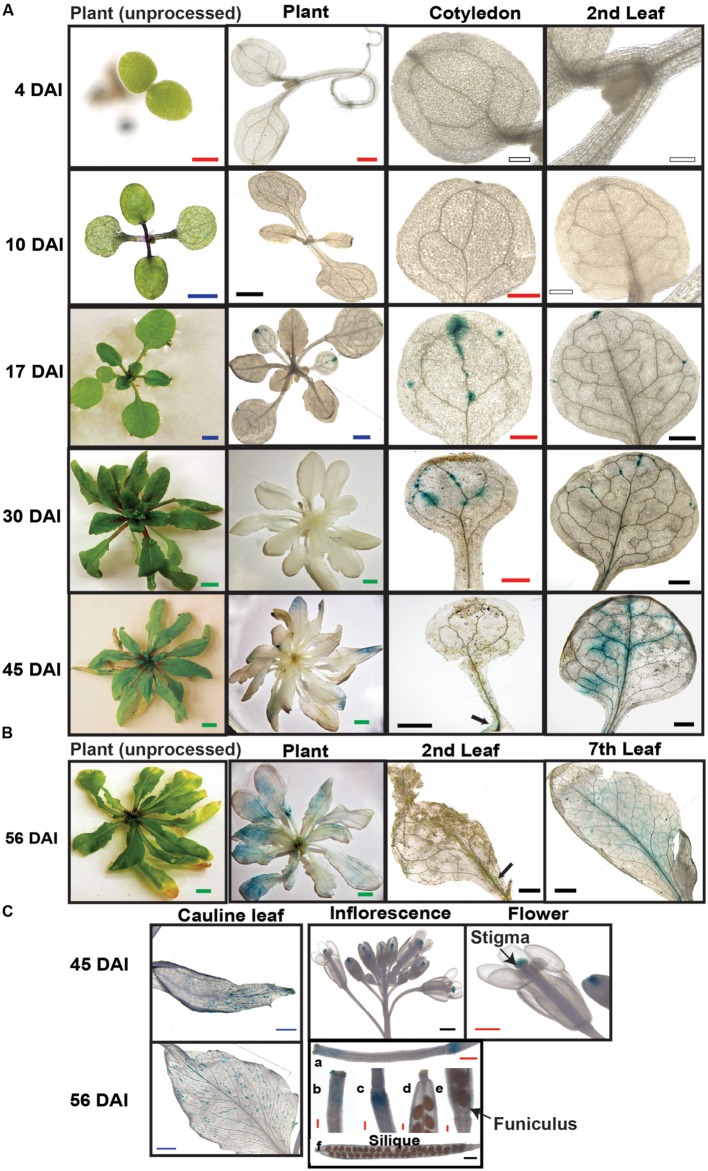
**Spatial and temporal expression of *SAQR*.** Beta-glucuronidase activity was visualized in transgenic *Arabidopsis* lines containing *SAQR* promoter::*GUS*. **(A)** *SAQR* expression in cotyledon, and first true leaf at 4, 10, 17, 30, and 45 DAI. **(B)** *SAQR* expression in 56-DAI plants in second leaf and seventh leaf. **(C)** Cauline leaf at 45 and 56 DAI; inflorescence and flower in 45-DAI plants; siliques, stigmas, and receptacle at 5 days after flowering (DAF; a-c) and 12 DAF (d-f). White bar, 200 μm; Red bar, 500 μm; Black bar, 1 mm; Blue bar, 2 mm; Green bar, 5 mm.

To evaluate the spatial and temporal changes in *SAQR* expression during development, we fused the *SAQR* promoter into a construct containing the GUS tag and introduced the construct into the *Arabidopsis* Col-0 background (**Figure 1B**; *SAQR::GUS* lines). *SAQR* is expressed in the vasculature of the regions of leaves and cotyledons that are approaching senescence, and continues to increase during senescence, then reducing as the cells die (**Figure 3A**). *SAQR* expression is detected at the tips of the leaves, is strongest in the vasculature as senescence progresses, and ends in the petiole. No *SAQR* expression was observed in young growing tissues. No *SAQR* expression was detected in the root at any stage of development (not shown). In 45-DAI plants, the older leaves express *SAQR* toward the apical end, which is where senescence first occurs. In 56-DAI plants, a stage of the *Arabidopsis* lifecycle in which most leaves are senescing, *SAQR* expression localizes progressively from the distal to proximal portions of the leaf as these sections die (**Figure 3B**). *SAQR* is also expressed in aging cauline leaves, and stigma of flowers, funiculus and receptacle of siliques (**Figure 3C**).

The analysis of SAQR promoter::GUS lines is consistent with, and expands on, the *SAQR* expression profile analysis. Specifically, many of the genes in the vasculature regulon (#57) are co-expressed with *SAQR* (**Table 1**) and *SAQR* expression is localized to the vasculature (**Figure 3**). Also, older leaves (and older regions of leaves) contain higher levels of *SAQR* transcript (**Figure 2**).

### *SAQR* is Induced under Specific Senescence Conditions

The increased *SAQR* transcript in senescence-related mutants of JA, ethylene, and ABA synthesis and signaling (**Figure 2A**), and the stress-related binding motifs in the *SAQR* promoter (**Supplementary Table S1**), implies that *SAQR* might be regulated by these hormones.

**FIGURE 4:**
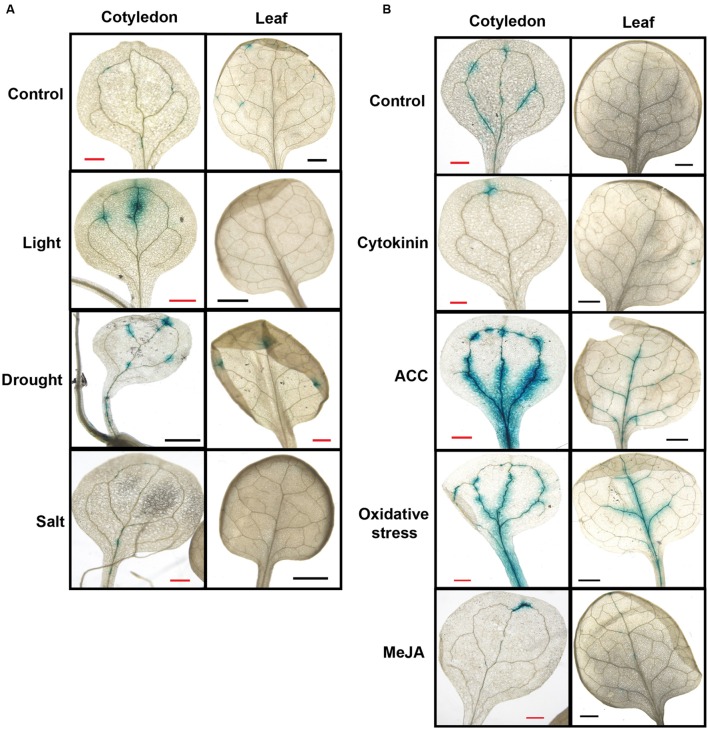
***Senescence-Associated and QQS-Related* expression is altered by diverse stresses.** Beta-glucuronidase activity was visualized in transgenic *Arabidopsis* lines containing *SAQR* promoter::*GUS*. **(A)** *SAQR* expression in cotyledon and true leaf of seedlings under environmental stresses. Seedlings grown in soil under constant light to 15 DAI (Control); seedlings grown in soil under constant light for 7 days, placed in the dark for 5 days, and exposed to light for 3 days (Light); seedlings grown to 15 DAI in soil under constant light in unwatered pots (Drought); and seedlings grown to 12 DAI in soil under constant light and then treated for 4 days by watering with 200 mM NaCl (Salt). **(B)** *SAQR* expression in cotyledon and true leaf of seedlings under chemical treatments. Seedlings grown in soil under constant light to 12 DAI were moved and placed for 4 days under constant light in: water (Control), or water plus 1 μM kinetin (Cytokinin), 50 μM of the ethylene precursor 1-aminocyclopropane-1-carboxylic acid (ACC), 500 μM hydrogen peroxide (Oxidative stress), or 10 μM methyl jasmonate (MeJA).

To investigate which conditions of senescence might increase *SAQR* expression and to identify the spatial patterns of expression, we examined patterns of *SAQR*-promoter-driven GUS expression under induced senescence (**Figure 4**). Because various methods of inducing senescence activate different genes (van der Graaff et al., 2006), we used three diverse methods to induce senescence. (1) Young seedlings were placed in darkness for 5 days and then exposed to constant light for 3 days (“light stress”; Weaver and Amasino, 2001). (2) Fully expanded true leaves attached to the plant were covered for 3 days (“dark stress”). (3) Fully expanded true leaves were detached and floated in water in the dark for 3 days (“dark stress of detached leaves”; van der Graaff et al., 2006).

Under the light stress, *SAQR* expression was increased in cotyledons but was reduced in true leaves compared to untreated controls (**Figure 4A**, Light). We also tested the effects of three different stresses – high salt, oxidative stress, and drought – on *SAQR* expression. Plant responses differed depending on the stress. Seedlings treated with NaCl did not show *SAQR* expression (**Figure 4A**, Salt) even though the plants are visibly damaged by the treatment (not shown). In contrast, seedlings dried to wilting (**Figure 4A**, Drought) or those treated with hydrogen peroxide (**Figure 4B**, Oxidative stress) show increased *SAQR* expression in the vasculature of the cotyledon and leaf.

*Senescence-Associated and QQS-Related* responds to different senescence-associated hormones. Seedlings treated with the artificial cytokinin (CK), kinetin, predictably were greener and showed reduced *SAQR* expression (**Figure 4B**, Cytokinin). Methyl JA treatments also showed reduced *SAQR* expression (**Figure 4B**, MeJA), whereas the JA-signaling *coi1* mutant showed increased *SAQR* expression (**Figure 2A**). In contrast, treatment with ethylene precursor increases senescence of the plant tissue noticeably more than the control, and results in significant increases in *SAQR* expression in the vasculature (**Figure 4B**, ACC).

Dark stress induces senescence in mature leaves, although somewhat different group of genes are expressed under dark stress compared to natural senescence (van der Graaff et al., 2006). Interestingly, *SAQR* expression did not change after either dark treatment (data not shown).

These results imply that *SAQR* is a SAG that responds to specific developmental signals coupled with environmental cues. The pattern of *SAQR* expression from germination to maturity implies that *SAQR* is up-regulated under natural senescence of cotyledons and true leaves.

### RNA-Seq of *SAQR* Knockout Line

To observe changes in gene expression associated with altered *SAQR* expression, we sequenced the RNA of rosette leaves of the SAQR KO line *saqr* and WT controls grown in a randomized complete block design under LD conditions and harvested at the end of the light cycle. The *saqr* line SALK_052233C contains a T-DNA insertion in the *SAQR* gene sequence and did not accumulate detectable *SAQR* RNA (**Supplementary Figure S2**). We also ordered a second putative *SAQR* T-DNA line, SALK_063861, from ABRC, but were unable to confirm the insertion in the genome. In addition to the expected decreased expression of *SAQR* (**Supplementary Table S7**), only two other genes were significantly differentially regulated when false discovery rate was controlled at 0.13 (**Supplementary Table S7**). These were a dirigent-like encoding gene (AT1G22900) and *ELIP1* (AT3G22840; EARLY LIGHT INDUCED PROTEIN 1).

AT1G22900, which encodes a disease-responsive protein that is a rather distant member of the dirigent family, was up-regulated sevenfold in the *saqr* mutant. The “dirigent” annotation implies an element that controls conformational chemistry (Burlat et al., 2001); the AT1G22900 protein has 32% identity to AT2G28670, which is required for correct localization of suberin (Hosmani et al., 2013). AT1G22900 is expressed in leaves at a low level under standard growth conditions; however, its expression is increased in response to plant exposure to *Pseudomonas syringae* (Schmid et al., 2005) and is suppressed in response to ABA treatment of plants exposed to *P. syringae* (Mohr and Cahill, 2006). Thus, both *SAQR* and AT1G22900 appear to be involved in stress responses, and AT1G22900 expression may be suppressed by *SAQR*.

*ELIP1* is decreased 3.6-fold in the *saqr* mutant. *ELIP1* is a member of the chlorophyll binding protein family and controls free chlorophyll levels (Hutin et al., 2003; Casazza et al., 2005; Yao et al., 2015). *ELIP1* is expressed highly in young plants, seeds and flowers. *ELIP1* has a protective role under UV-B and photosensitive stress in high light or cold (Hutin et al., 2003). It is up-regulated quickly and transiently by light including UV-B, and is up-regulated under a variety of stresses including *P. syringae* infection (Hutin et al., 2003; Rossini et al., 2006; Hruz et al., 2008); in spruce ELP-like proteins are induced by weevil and western spruce budworm infection (Ralph et al., 2007).

### Phenotypic Characterization of *SAQR* T-DNA Knockout and Transgenic Overexpression Lines

**FIGURE 5:**
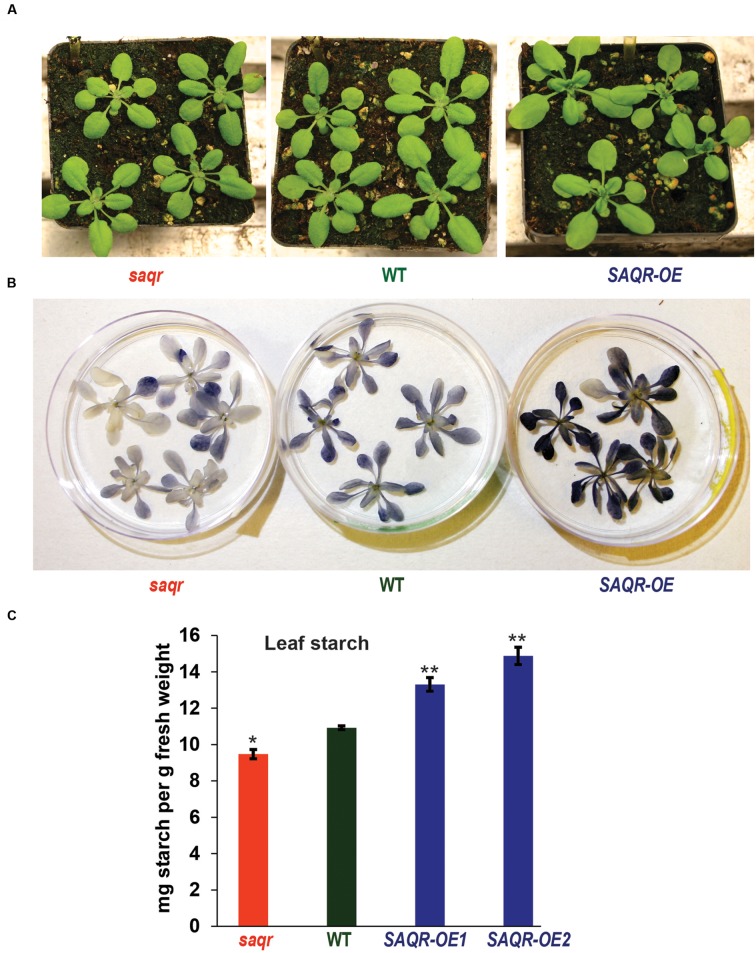
**Starch content of *SAQR* knockout (KO) and overexpression lines.** **(A)** *KO* and *OE* lines are visually similar to WT controls. **(B)** Qualitative starch staining shows increased starch in *SAQR-OE* lines and decreased starch in *saqr* compared with WT. **(C)** Quantification of leaf starch levels. Data points are the mean ± SEM (standard error of the mean) of three biological replicates, with five plants per replicate. The *saqr* mutant, WT control and *OE* mutant plants were grown in a completely randomized design in the soil in pots under LD conditions, and harvested for starch determination at the end of the light period. Single-factor analysis of variance (ANOVA) with Dunnett’s method was used to compare each mutant with WT. ^∗^*P* < 0.05, ^∗∗^*P* < 0.01.

**FIGURE 6:**
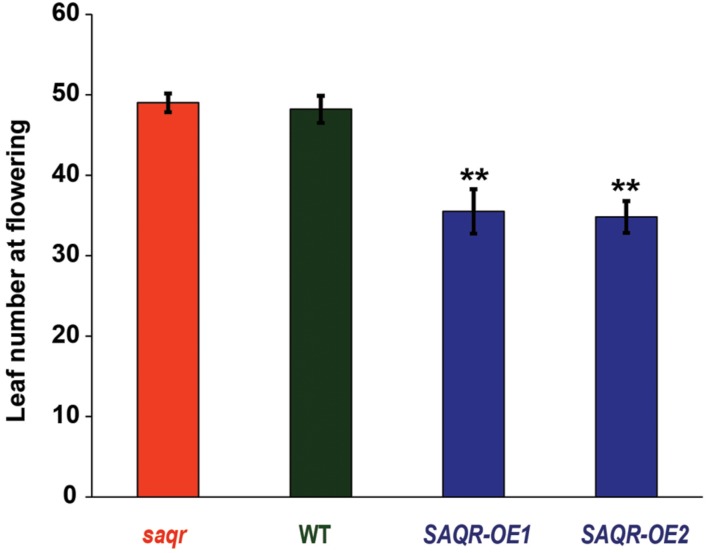
***Senescence-Associated and QQS-Related* overexpression mutants have fewer leaves upon flowering.** *SAQR* KO mutant, WT control, and *OE* mutant plants were grown in a completely randomized design under SD conditions. Bars represent mean ± SEM of rosette leaf count of four to six biological replicate plants upon flowering. Single-factor analysis of variance (ANOVA) with Dunnett’s method was used to compare each mutant with WT. ^∗^*P* < 0.05, ^∗∗^*P* < 0.01.

Taken together, our data indicates that *SAQR* plays a role in stress resistance. To directly investigate the function of *SAQR* in *Arabidopsis*, we generated *SAQR-OE* lines driven by the 35S promoter (**Figure 1B**). The OE plants were verified for curtailed *SAQR* expression by semi-quantitative RT-PCR (**Supplementary Figure S2**). When grown under constant light or under LD conditions the KO and OE lines appear phenotypically similar to Col-0 control plants (**Figure 5A**). The *SAQR-OE* lines show an early-flowering phenotype (also in Luhua et al., 2008), and fewer leaves are required for flowering (**Figure 6**). However, *saqr* plants do not show any difference in flowering time when grown under SD conditions (**Figure 6**). When plants are treated with salt, cytokinin, or ACC, *saqr* and *SAQR-OE* mutants show a similar visual phenotype to the WT controls (**Supplementary Figure S4**).

Because alteration of *QQS* changes starch biosynthesis and accumulation, and *SAQR* expression is up-regulated in *QQS* RNAi lines, but *QQS* does not have significantly altered accumulation of transcript in *saqr* mutant, we proposed that *SAQR* might act downstream of *QQS*. Therefore, we evaluated leaf starch in mutants with altered accumulation of *SAQR*. At the end of the light cycle in plants grown in a completely randomized design under LD conditions, leaf starch content is decreased about 13% in the *saqr* mutant and increased about 20–35% in the *SAQR-OE* lines, when compared to WT plants (**Figures 5B,C**). Thus, permutations in *SAQR* expression strongly impact starch accumulation.

## Discussion

Senescence in plants can be defined as the cellular signaling program that leads to the degeneration and eventual death of tissue. More than simply the process of aging affecting the plant, senescence is a process that is triggered by various internal and external factors and that serves to recycle nutrients and manage exposure to stresses (Noodén et al., 1997; Lim et al., 2007).

Senescence is precisely induced and regulated by development, hormones, darkness, nutrient limitation, damage by pathogens and abiotic environmental stresses (Noodén et al., 1997; Lim et al., 2007; Liang et al., 2014). It has been proposed that this tightly controlled process evolved to secure maximal nutrient efficiency under limiting conditions (Leopold, 1961; Masclaux-Daubresse et al., 2008). During senescence, anabolic processes like photosynthesis and metabolite synthesis are reduced (Buchanan-Wollaston et al., 2003), while multiple molecular components undergo controlled degradation for transport through the phloem to the rest of the plant (Thompson et al., 1998; Liu et al., 2008).

In *Arabidopsis*, each leaf has its own timeline of expansion, maturity, and senescence, independent of the reproductive stage of the plant; the development occurs despites the removal or disruption of flowering tissue, with reproductive factors only effecting individual leaves in the context of a separate, whole plant-scale program of senescence (Noodén and Penney, 2001; Lim et al., 2007). Under controlled, optimized conditions, each leaf grows from a vegetative meristem, through division and cellular expansion. Cells transition from division to expansion starting from the tip of the leaf (Andriankaja et al., 2012; Gonzalez et al., 2012). The leaf reaches full photosynthetic activity by approximately 12 days and visible senescence begins approximately 20–24 days after emergence. The yellowing of the leaf and transfer of nutrients via the vascular system begins at this stage, again proceeding from the tip to the base of the leaf. The localization of *SAQR* expression in the vasculature and its timing from the tip to the base of the leaf increasing just prior to the onset of senescence, indicates a possible involvement of this gene in nutrient recycling.

The final, destructive process, generally termed leaf “death,” occurs 28–32 days after the leaf’s initial emergence (Lim et al., 2007). The speed of this process is controlled by light dosage, individual leaves exposed to decreased light levels show increased senescence (Nooden et al., 1996; Weaver and Amasino, 2001). As the reproductive program commences, rosette leaf formation ceases, and nutrient allocation is shifted to the growing reproductive structures. Increased metabolic activity in mitochondria and peroxisomes, and decreased peroxisomal catalase and cytosolic ascorbate peroxidase (APX1) activities result in a spike in the production of reactive oxygen species (ROS; Beers, 1997; Zimmermann et al., 2006). This spike, coupled with a general decrease in general antioxidant activity, augments oxidative damage, and death ensues (Procházková and Wilhelmová, 2007).

**FIGURE 7:**
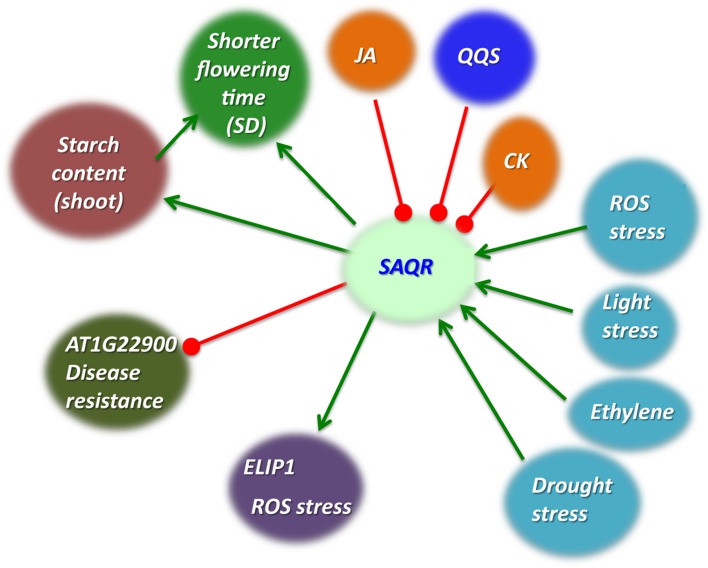
**Model of SAQR function.** Decreased *QQS* expression or increased stress cause greater starch accumulation. *SAQR* expression is up-regulated by multiple stresses and down-regulated by *QQS* (Li et al., 2015b), JA, and CK (data of **Figure 4**). Multiple stresses increase starch accumulation; QQS, MeJA (Babst et al., 2005) and CK decrease starch accumulation. *SAQR* expression increases starch accumulation. Our working model is that SAQR mediates changes in starch accumulation. *SAQR* expression also decreases time to flowering under short day conditions. This change in flowering time may occur via some aspect of starch metabolism, or by a mechanism independent of starch metabolism. Green arrows, promotes; red blocked lines, represses.

Although the molecular function of SAQR is unclear, there are several indications of its potential biological functions (**Figure 7**). Reflecting the patterns of senescence itself, during natural senescence, *SAQR* is up-regulated in the cotyledons and true leaves, whereas in light stress-induced senescence, *SAQR* expression is up-regulated only in the cotyledons, and is repressed in the true leaves. This is evidenced not only in the *SAQR* expression patterns, but also by the tight correlation of expression of many SAG genes to *SAQR*. Several factors might lead to this distinction between *SAQR* expression in true leaves and cotyledons. Cotyledon senescence is less understood than leaf senescence, but the processes have developmental and molecular differences (Du et al., 2014). Cotyledon senescence is induced by different signals than is true leaf senescence; it has been suggested that these differences are due to the cotyledon’s early function as a storage organ (Weaver and Amasino, 2001). Some sets of genes are differentially expressed in cotyledons compared to true leaves; many of the genes specific to or differentially expressed in soybean cotyledons are involved in early mobilization of nutrients, indicating a rapid transfer of resources to the seedling (Brown and Hudson, 2015). This mimics the nutrient transfer process that occurs under senescence of older leaves (Diaz et al., 2008). In fact, when plant are treated by light after dark, naturally senescing true leaves that are already undergoing transfer of nutrients to the rest of the plant exhibit less delay in senescence compared to younger leaves (Weaver and Amasino, 2001). The increase of *SAQR* expression in cotyledons under light stress likely reflects this difference between cotyledons and early true leaves.

The *maltose excess 1* (*mex1*) mutant is a null mutant of a chloroplastic maltose transporter (Lu et al., 2006; Stettler et al., 2009). Young leaves of *mex1* plants have increased maltose and starch and show signs of chloroplast degradation relative to WT plants (Stettler et al., 2009); increased maltose and starch levels and chloroplast degradation are general characteristics of mature/senescing leaves of WT (and *mex1*) plants (Stettler et al., 2009; Avila-Ospina et al., 2014). Analysis of the transcriptomic data from Stettler et al. (2009) shows the *SAQR* transcript level is increased 3.3-fold in young leaves (leaf # 6–8) in *mex1* relative to WT, whereas in mature leaves (leaf # 13–15) *SAQR* transcript level is not significantly different in *mex1* and WT plants. These data indicate that *SAQR* may be related to senescence, be sensitive to changes in carbohydrate metabolism, while also playing a role in reducing starch content, as can be seen from *SAQR-OE* plants (**Figure 7**).

Although *SAQR* influences starch levels, alterations in the expression of *SAQR* do not cause a notable difference in the rate or severity of leaf senescence in plants grown under standard conditions. This is not entirely unexpected, as genes up-regulated under senescence have many functions: transcription factors, catabolic enzymes (*e.g.*, proteases and kinases), or signaling and structural functions. Many SAG genes may not directly affect senescence. For example, senescence is unaffected in homozygous mutants of the cysteine protease encoding gene *SAG12* (Otegui et al., 2005).

*Senescence-Associated and QQS-Related* is one of the class of mobile RNAs which are translocated in the plant (Thieme et al., 2015). This mobility may provide a clue about its mechanism of action. *SAQR* expression is predominantly confined to the vasculature during early and mid-senescence, and its mobility may explain how it induces the overall increase in starch that can be seen in leaves of *SAQR-OE* plants. Similarly, the detection of two binding sites of AGL15 – overexpression of which delays flowering and senescence (Fang and Fernandez, 2002) – in *SAQR* promoter, alterations in *SAQR* impact flowering time, with overexpression of *SAQR* inducing an early flowering phenotype (Luhua et al., 2008, and this study), combined with the location and timing of *SAQR* expression, indicates that *SAQR* could play a role as a mobile messenger in flowering.

## Conclusion

*Senescence-Associated and QQS-Related* is a clade-specific gene, present in three closely related *Brassicaceae* genera. One role of such clade-specific genes is thought to be the adaptation of plants to stress (Luhua et al., 2013; Arendsee et al., 2014). In this study, we present *SAQR* as a component of the interconnected networks integrating stress signaling, metabolism, and senescence. *SAQR* is up-regulated in *QQS* RNAi mutant lines, and *QQS* expression negatively affects starch levels (Li et al., 2009; Li and Wurtele, 2015). Alterations in the expression of *SAQR* change levels of starch accumulation, but *QQS* expression is not altered in the KO mutant. Taken together, these results indicate that *SAQR* may participate in the *QQS* network, downstream of *QQS*. *SAQR* is up-regulated under conditions of natural senescence, and is co-expressed with genes involved in senescence, defense, and stress responses, implying a complex role in the interplay between primary metabolism and adaptation to the stresses that occur alongside the process of senescence.

These analyses of SAQR function provide a clue as to the mechanisms by which plants integrate metabolism with natural and environmentally induced senescence, advancing our fundamental knowledge of the regulatory and metabolic network that mediates carbon allocation. The data also inform the current view of the evolutionary significance of clade-specific genes. A number of proteins encoded by orphans and other clade-specific genes have defined functions (Cai et al., 2008; Heinen et al., 2009; Knowles and McLysaght, 2009; Li et al., 2009, 2010), and the QQS gene itself interacts with highly conserved proteins and thus can function in multiple plant species (Li et al., 2015b). If this is the case for SAQR, these studies provide an avenue to the potential use of this gene to modulate stress adaptation and/or composition for economically valuable crop plants.

## Author Contributions

DJ and LL designed the research; DJ, WZ, SH, RY, and LL performed the research; DJ, XZ, CD, DN, RY, TS, and LL analyzed the data; DJ, DN, EW, and LL wrote the paper.

## Conflict of Interest Statement

The authors declare that the research was conducted in the absence of any commercial or financial relationships that could be construed as a potential conflict of interest.

## Footnotes

1 https://abrc.osu.edu/

2 http://www.metnetdb.org/MetNet_MetaOmGraph.htm.

3 http://bioinformatics.psb.ugent.be/webtools/plantcare/html/

4 http://bgi.com/us/

5 https://www.ncbi.nlm.nih.gov/sra/

6 http://www.plantcyc.org/databases/aracyc/14.0

7 http://agris.fao.org/agris-search/index.do

8 http://www.metnetdb.org/MetNet_db.htm

## References

[B1] Allu A. D. Soja A. M. Wu A. Szymanski J. Balazadeh S. (2014). Salt stress and senescence: identification of cross-talk regulatory components. *J. Exp. Bot.* 65 3993–4008. 10.1093/jxb/eru173 24803504 PMC4106443

[B2] Andriankaja M. Dhondt S. De bodt S. Vanhaeren H. Coppens F. De milde L. (2012). Exit from proliferation during leaf development in *Arabidopsis thaliana*: a not-so-gradual process. *Dev. Cell* 22 64–78. 10.1016/j.devcel.2011.11.011 22227310

[B3] Arendsee Z. W. Li L. Wurtele E. S. (2014). Coming of age: orphan genes in plants. *Trends Plant Sci.* 19 698–708. 10.1016/j.tplants.2014.07.003 25151064

[B4] Avila-Ospina L. Moison M. Yoshimoto K. Masclaux-Daubresse C. (2014). Autophagy, plant senescence, and nutrient recycling. *J. Exp. Bot.* 65 3799–3811. 10.1093/jxb/eru039 24687977

[B5] Babst B. A. Ferrieri R. A. Gray D. W. Lerdau M. Schlyer D. J. Schueller M. (2005). Jasmonic acid induces rapid changes in carbon transport and partitioning in *Populus*. *New Phytol.* 167 63–72. 10.1111/j.1469-8137.2005.01388.x 15948830

[B6] Battaglia M. Olvera-Carrillo Y. Garciarrubio A. Campos F. Covarrubias A. A. (2008). The enigmatic LEA proteins and other hydrophilins. *Plant Physiol.* 148 6–24. 10.1104/pp.108.120725 18772351 PMC2528095

[B7] Beaudoin N. Serizet C. Gosti F. Giraudat J. (2000). Interactions between abscisic acid and ethylene signaling cascades. *Plant Cell* 12 1103–1115. 10.2307/3871258 10899977 PMC149052

[B8] Beers E. P. (1997). Programmed cell death during plant growth and development. *Cell Death Differ.* 4 649–661. 10.1038/sj.cdd.4400297 16465277

[B9] Borg M. Brownfield L. Khatab H. Sidorova A. Lingaya M. Twell D. (2011). The R2R3 MYB transcription factor DUO1 activates a male germline-specific regulon essential for sperm cell differentiation in *Arabidopsis*. *Plant Cell* 23 534–549. 10.1105/tpc.110.081059 21285328 PMC3077786

[B10] Breeze E. Harrison E. Mchattie S. Hughes L. Hickman R. Hill C. (2011). High-resolution temporal profiling of transcripts during *Arabidopsis* leaf senescence reveals a distinct chronology of processes and regulation. *Plant Cell* 23 873–894. 10.1105/tpc.111.083345 21447789 PMC3082270

[B11] Brown A. V. Hudson K. A. (2015). Developmental profiling of gene expression in soybean trifoliate leaves and cotyledons. *BMC Plant Biol.* 15:169 10.1186/s12870-015-0553-y PMC4492100 26149852

[B12] Buchanan-Wollaston V. Earl S. Harrison E. Mathas E. Navabpour S. Page T. (2003). The molecular analysis of leaf senescence–a genomics approach. *Plant Biotechnol. J.* 1 3–22. 10.1046/j.1467-7652.2003.00004.x 17147676

[B13] Buchanan-Wollaston V. Page T. Harrison E. Breeze E. Lim P. O. Nam H. G. (2005). Comparative transcriptome analysis reveals significant differences in gene expression and signalling pathways between developmental and dark/starvation-induced senescence in *Arabidopsis*. *Plant J.* 42 567–585. 10.1111/j.1365-313X.2005.02399.x 15860015

[B14] Burlat V. Kwon M. Davin L. B. Lewis N. G. (2001). Dirigent proteins and dirigent sites in lignifying tissues. *Phytochemistry* 57 883–897. 10.1016/S0031-9422(01)00117-0 11423139

[B15] Cai J. Zhao R. Jiang H. Wang W. (2008). De novo origination of a new protein-coding gene in *Saccharomyces cerevisiae*. *Genetics* 179 487–496. 10.1534/genetics.107.084491 18493065 PMC2390625

[B16] Casazza A. Rossini S. Rosso M. Soave C. (2005). Mutational and expression analysis of ELIP1 and ELIP2 in *Arabidopsis thaliana*. *Plant Mol. Biol.* 58 41–51. 10.1007/s11103-005-4090-1 16028115

[B17] Clauss M. J. Koch M. A. (2006). Poorly known relatives of *Arabidopsis thaliana*. *Trends Plant Sci.* 11 449–459. 10.1016/j.tplants.2006.07.005 16893672

[B18] Clough S. J. Bent A. F. (1998). Floral dip: a simplified method for *Agrobacterium*-mediated transformation of *Arabidopsis thaliana*. *Plant J.* 16 735–743. 10.1046/j.1365-313x.1998.00343.x 10069079

[B19] Coenen C. Lomax T. L. (1998). The diageotropica gene differentially affects auxin and cytokinin responses throughout development in tomato. *Plant Physiol.* 117 63–72. 10.1104/pp.117.1.63 9576775 PMC35022

[B20] Davletova S. Rizhsky L. Liang H. Shengqiang Z. Oliver D. J. Coutu J. (2005). Cytosolic ascorbate peroxidase 1 is a central component of the reactive oxygen gene network of *Arabidopsis*. *Plant Cell* 17 268–281. 10.1105/tpc.104.026971 15608336 PMC544504

[B21] de Marcos A. Triviño M. Pérez-Bueno M. L. Ballesteros I. Barón M. Mena M. (2015). Transcriptional profiles of *Arabidopsis* stomataless mutants reveal developmental and physiological features of life in the absence of stomata. *Front. Plant Sci.* 6:456 10.3389/fpls.2015.00456 PMC4477074 26157447

[B22] DeLano W. L. Bromberg S. (2002). *The PyMOL User’s Manual.* Palo Alto, CA: DeLano Scientific.

[B23] Devoto A. Turner J. G. (2003). Regulation of jasmonate-mediated plant responses in *Arabidopsis*. *Ann. Bot.* 92 329–337. 10.1093/aob/mcg151 12871847 PMC4257513

[B24] Diaz C. Lemaître T. Christ A. Azzopardi M. Kato Y. Sato F. (2008). Nitrogen recycling and remobilization are differentially controlled by leaf senescence and development stage in *Arabidopsis* under low nitrogen nutrition. *Plant Physiol.* 147 1437–1449. 10.1104/pp.108.119040 18467460 PMC2442554

[B25] Domazet-Lošo T. Brajković J. Tautz D. (2007). A phylostratigraphy approach to uncover the genomic history of major adaptations in metazoan lineages. *Trends Genet.* 23 533–539. 10.1016/j.tig.2007.08.014 18029048

[B26] Du J. Li M. Kong D. Wang L. Lv Q. Wang J. (2014). Nitric oxide induces cotyledon senescence involving co-operation of the NES1/MAD1 and EIN2-associated ORE1 signalling pathways in *Arabidopsis*. *J. Exp. Bot.* 65 4051–4063. 10.1093/jxb/ert429 24336389 PMC4106434

[B27] Eom J. Baker W. R. Kintanar A. Wurtele E. S. (1996). The embryo-specific EMB-1 protein of *Daucus carota* is flexible and unstructured in solution. *Plant Sci.* 115 17–24. 10.1016/0168-9452(96)04332-4

[B28] Fang S.-C. Fernandez D. E. (2002). Effect of regulated overexpression of the MADS domain factor AGL15 on flower senescence and fruit maturation. *Plant Physiol.* 130 78–89. 10.1104/pp.004721 12226488 PMC166541

[B29] Gollery M. Harper J. Cushman J. Mittler T. Girke T. Zhu J.-K. (2006). What makes species unique? The contribution of proteins with obscure features. *Genome Biol.* 7:R57 10.1186/gb-2006-7-7-r57 PMC1779552 16859532

[B30] Gonzalez N. Vanhaeren H. Inzé D. (2012). Leaf size control: complex coordination of cell division and expansion. *Trends Plant Sci.* 17 332–340. 10.1016/j.tplants.2012.02.003 22401845

[B31] He Y. Fukushige H. Hildebrand D. F. Gan S. (2002). Evidence supporting a role of jasmonic acid in *Arabidopsis* leaf senescence. *Plant Physiol.* 128 876–884. 10.1104/pp.010843 11891244 PMC152201

[B32] Heinen T. J. Staubach F. Haming D. Tautz D. (2009). Emergence of a new gene from an intergenic region. *Curr. Biol.* 19 1527–1531. 10.1016/j.cub.2009.07.049 19733073

[B33] Horan K. Jang C. Bailey-Serres J. Mittler R. Shelton C. Harper J. F. (2008). Annotating genes of known and unknown function by large-scale coexpression analysis. *Plant Physiol.* 147 41–57. 10.1104/pp.108.117366 18354039 PMC2330292

[B34] Hosmani P. S. Kamiya T. Danku J. Naseer S. Geldner N. Guerinot M. L. (2013). Dirigent domain-containing protein is part of the machinery required for formation of the lignin-based Casparian strip in the root. *Proc. Natl. Acad. Sci. U.S.A.* 110 14498–14503. 10.1073/pnas.1308412110 23940370 PMC3761638

[B35] Hruz T. Laule O. Szabo G. Wessendorp F. Bleuler S. Oertle L. (2008). Genevestigator V3: a reference expression database for the meta-analysis of transcriptomes. *Adv. Bioinform.* 2008:420747 10.1155/2008/420747 PMC2777001 19956698

[B36] Hutin C. Nussaume L. Moise N. Moya I. Kloppstech K. Havaux M. (2003). Early light-induced proteins protect *Arabidopsis* from photooxidative stress. *Proc. Natl. Acad. Sci. U.S.A.* 100 4921–4926. 10.1073/pnas.0736939100 12676998 PMC153656

[B37] Jing H.-C. Anderson L. Sturre M. J. G. Hille J. Dijkwel P. P. (2007). *Arabidopsis* CPR5 is a senescence-regulatory gene with pleiotropic functions as predicted by the evolutionary theory of senescence. *J. Exp. Bot.* 58 3885–3894. 10.1093/jxb/erm237 18033818

[B38] Knowles D. G. McLysaght A. (2009). Recent de novo origin of human protein-coding genes. *Genome Res.* 19 1752–1759. 10.1101/gr.095026.109 19726446 PMC2765279

[B39] Koch M. A. Kiefer M. (2005). Genome evolution among cruciferous plants: a lecture from the comparison of the genetic maps of three diploid species—*Capsella rubella*, *Arabidopsis lyrata* subsp. *petraea*, and *A. thaliana*. *Am. J. Bot.* 92 761–767. 10.3732/ajb.92.4.761 21652456

[B40] Koornneef M. Jorna M. Brinkhorst-Van Der Swan D. Karssen C. (1982). The isolation of abscisic acid (ABA) deficient mutants by selection of induced revertants in non-germinating gibberellin sensitive lines of *Arabidopsis thaliana* (L.) Heynh. *Theor. Appl. Genet.* 61 385–393. 10.1007/BF00272861 24270501

[B41] Kozlowski L. P. Bujnicki J. M. (2012). MetaDisorder: a meta-server for the prediction of intrinsic disorder in proteins. *BMC Bioinformatics* 13:111 10.1186/1471-2105-13-111 PMC3465245 22624656

[B42] Lamesch P. Berardini T. Z. Li D. H. Swarbreck D. Wilks C. Sasidharan R. (2012). The *Arabidopsis* information resource (TAIR): improved gene annotation and new tools. *Nucleic Acids Res.* 40 D1202–D1210. 10.1093/nar/gkr1090 22140109 PMC3245047

[B43] Leopold A. C. (1961). Senescence in plant development. *Science* 134 1727–1732. 10.2307/1707929 17779068

[B44] Li D. Dong Y. Jiang Y. Jiang H. Cai J. Wang W. (2010). A de novo originated gene depresses budding yeast mating pathway and is repressed by the protein encoded by its antisense strand. *Cell Res.* 20 408–420. 10.1038/cr.2010.31 20195295

[B45] Li L. Foster C. M. Gan Q. Nettleton D. James M. G. Myers A. M. (2009). Identification of the novel protein QQS as a component of the starch metabolic network in *Arabidopsis* leaves. *Plant J.* 58 485–498. 10.1111/j.1365-313X.2009.03793.x 19154206

[B46] Li L. Hur M. Lee J.-Y. Zhou W. Song Z. Ransom N. (2015a). A systems biology approach toward understanding seed composition in soybean. *BMC Genomics* 16:S9 10.1186/1471-2164-16-S3-S9 PMC4331812 25708381

[B47] Li L. Ilarslan H. James M. G. Myers A. M. Wurtele E. S. (2007). Genome wide co-expression among the starch debranching enzyme genes AtISA1 AtISA2 and AtISA3 in *Arabidopsis thaliana*. *J. Exp. Bot.* 58 3323–3342. 10.1093/jxb/erm180 17890231

[B48] Li L. Wurtele E. S. (2015). The QQS orphan gene of *Arabidopsis* modulates carbon and nitrogen allocation in soybean. *Plant Biotechnol. J.* 13 177–187. 10.1111/pbi.12238 25146936 PMC4345402

[B49] Li L. Zheng W. Zhu Y. Ye H. Tang B. Arendsee Z. W. (2015b). QQS orphan gene regulates carbon and nitrogen partitioning across species via NF-YC interactions. *Proc. Natl. Acad. Sci. U.S.A.* 112 14734–14739. 10.1073/pnas.1514670112 26554020 PMC4664325

[B50] Liang C. Wang Y. Zhu Y. Tang J. Hu B. Liu L. (2014). OsNAP connects abscisic acid and leaf senescence by fine-tuning abscisic acid biosynthesis and directly targeting senescence-associated genes in rice. *Proc. Natl. Acad. Sci. U.S.A.* 111 10013–10018. 10.1073/pnas.1321568111 24951508 PMC4103337

[B51] Lim P. O. Kim H. J. Nam H. G. (2007). Leaf senescence. *Annu. Rev. Plant Biol.* 58 115–136. 10.1146/annurev.arplant.57.032905.105316 17177638

[B52] Liu J. Wu Y. Yang J. Liu Y. Shen F. (2008). Protein degradation and nitrogen remobilization during leaf senescence. *J. Plant Biol.* 51 11–19. 10.1007/BF03030735

[B53] López-Martínez G. Rodríguez-Porrata B. Margalef-Català M. Cordero-Otero R. (2012). The STF2p hydrophilin from *Saccharomyces cerevisiae* is required for dehydration stress tolerance. *PLoS ONE* 7:e33324 10.1371/journal.pone.0033324 PMC3306391 22442684

[B54] Lu Y. Steichen J. M. Weise S. E. Sharkey T. D. (2006). Cellular and organ level localization of maltose in maltose-excess *Arabidopsis* mutants. *Planta* 224 935–943. 10.1007/s00425-006-0263-7 16596410

[B55] Luhua S. Ciftci-Yilmaz S. Harper J. Cushman J. Mittler R. (2008). Enhanced tolerance to oxidative stress in transgenic *Arabidopsis* plants expressing proteins of unknown function. *Plant Physiol.* 148 280–292. 10.1104/pp.108.124875 18614705 PMC2528079

[B56] Luhua S. Hegie A. Suzuki N. Shulaev E. Luo X. Z. Cenariu D. (2013). Linking genes of unknown function with abiotic stress responses by high-throughput phenotype screening. *Physiol. Plant.* 148 322–333. 10.1111/ppl.12013 23517122

[B57] Masclaux-Daubresse C. Reisdorf-Cren M. Orsel M. (2008). Leaf nitrogen remobilisation for plant development and grain filling. *Plant Biol.* 10 23–36. 10.1111/j.1438-8677.2008.00097.x 18721309

[B58] Mentzen W. I. Peng J. Ransom N. Nikolau B. J. Wurtele E. S. (2008). Articulation of three core metabolic processes in *Arabidopsis*: fatty acid biosynthesis, leucine catabolism and starch metabolism. *BMC Plant Biol.* 8:76 10.1186/1471-2229-8-76 PMC2483283 18616834

[B59] Mentzen W. I. Wurtele E. S. (2008). Regulon organization of *Arabidopsis*. *BMC Plant Biol.* 8:99 10.1186/1471-2229-8-99 PMC2567982 18826618

[B60] Miao H. Wei J. Zhao Y. Yan H. Sun B. Huang J. (2013). Glucose signalling positively regulates aliphatic glucosinolate biosynthesis. *J. Exp. Bot.* 64 1097–1109. 10.1093/jxb/ers399 23329848 PMC3580823

[B61] Mitchell-Olds T. Al-Shehbaz I. A. Koch M. Sharbel T. F. (2005). “Crucifer evolution in the post-genomic era,” in *Plant Diversity and Evolution: Genotypic and Phenotypic Variation in Higher Plants*, ed. Henry R. J. (Wallingford: CABI Publishing), 119–137. 10.1079/9780851999043.0119

[B62] Mohr P. G. Cahill D. M. (2006). Suppression by ABA of salicylic acid and lignin accumulation and the expression of multiple genes, in *Arabidopsis* infected with *Pseudomonas syringae* pv. tomato. *Funct. Integr. Genomics* 7 181–191. 10.1007/s10142-006-0041-4 17149585

[B63] Mueller-Roeber B. Balazadeh S. (2014). Auxin and its role in plant senescence. *J. Plant Growth Regul.* 33 21–33. 10.1007/s00344-013-9398-5

[B64] Mukaka M. M. (2012). A guide to appropriate use of correlation coefficient in medical research. *Malawi Med. J.* 24 69–71. 23638278 PMC3576830

[B65] Murray S. L. Thomson C. Chini A. Read N. D. Loake G. J. (2002). Characterization of a novel, defense-related *Arabidopsis* mutant, cir1 isolated by luciferase imaging. *Mol. Plant Microbe Interact.* 15 557–566. 10.1094/MPMI.2002.15.6.557 12059104

[B66] Neme R. Tautz D. (2013). Phylogenetic patterns of emergence of new genes support a model of frequent de novo evolution. *BMC Genomics* 14:117 10.1186/1471-2164-14-117 PMC3616865 23433480

[B67] Ngaki M. N. Louie G. V. Philippe R. N. Manning G. Pojer F. Bowman M. E. (2012). Evolution of the chalcone isomerase fold from fatty acid-binding to stereospecific enzyme. *Nature* 485 530–533. 10.1038/nature11009 22622584 PMC3880581

[B68] Niyogi K. K. Grossman A. R. Björkman O. (1998). *Arabidopsis* mutants define a central role for the xanthophyll cycle in the regulation of photosynthetic energy conversion. *Plant Cell* 10 1121–1134. 10.2307/3870716 9668132 PMC144052

[B69] Noodén L. D. Guiamét J. J. John I. (1997). Senescence mechanisms. *Physiol. Plant.* 101 746–753. 10.1111/j.1399-3054.1997.tb01059.x

[B70] Nooden L. D. Hillsberg J. W. Schneider M. J. (1996). Induction of leaf senescence in *Arabidopsis thaliana* by long days through a light-dosage effect. *Physiol. Plant.* 96 491–495. 10.1111/j.1399-3054.1996.tb00463.x

[B71] Noodén L. D. Penney J. P. (2001). Correlative controls of senescence and plant death in *Arabidopsis thaliana* (Brassicaceae). *J. Exp. Bot.* 52 2151–2159. 10.1093/jexbot/52.364.2151 11604454

[B72] O’Connor T. R. Dyreson C. Wyrick J. J. (2005). Athena: a resource for rapid visualization and systematic analysis of *Arabidopsis* promoter sequences. *Bioinformatics* 21 4411–4413. 10.1093/bioinformatics/bti714 16223790

[B73] Oh S. A. Park J. H. Lee G. I. Paek K. H. Park S. K. Nam H. G. (1997). Identification of three genetic loci controlling leaf senescence in *Arabidopsis thaliana*. *Plant J.* 12 527–535. 10.1046/j.1365-313X.1997.00489.x 9351240

[B74] Olvera-Carrillo Y. Reyes J. L. Covarrubias A. A. (2011). Late embryogenesis abundant proteins: versatile players in the plant adaptation to water limiting environments. *Plant Signal. Behav.* 6:586 10.4161/psb.6.4.15042 PMC3142399 21447997

[B75] Otegui M. S. Noh Y.-S. Martínez D. E. Vila Petroff M. G. Andrew Staehelin L. Amasino R. M. (2005). Senescence-associated vacuoles with intense proteolytic activity develop in leaves of *Arabidopsis* and soybean. *Plant J.* 41 831–844. 10.1111/j.1365-313X.2005.02346.x 15743448

[B76] Procházková D. Wilhelmová N. (2007). Leaf senescence and activities of the antioxidant enzymes. *Biol. Plant.* 51 401–406. 10.1007/s10535-007-0088-7

[B77] Ralph S. G. Jancsik S. Bohlmann J. (2007). Dirigent proteins in conifer defense II: extended gene discovery, phylogeny, and constitutive and stress-induced gene expression in spruce (*Picea* spp.). *Phytochemistry* 68 1975–1991. 10.1016/j.phytochem.2007.04.042 17590394

[B78] Reyes J. L. Rodrigo M.-J. Colmenero-Flores J. M. Gil J.-V. Garay-Arroyo A. Campos F. (2005). Hydrophilins from distant organisms can protect enzymatic activities from water limitation effects in vitro. *Plant Cell Environ.* 28 709–718. 10.1111/j.1365-3040.2005.01317.x

[B79] Rossini S. Casazza A. P. Engelmann E. C. Havaux M. Jennings R. C. Soave C. (2006). Suppression of both ELIP1 and ELIP2 in *Arabidopsis* does not affect tolerance to photoinhibition and photooxidative stress. *Plant Physiol.* 141 1264–1273. 10.1104/pp.106.083055 16778010 PMC1533944

[B80] Roy A. Kucukural A. Zhang Y. (2010). I-TASSER: a unified platform for automated protein structure and function prediction. *Nat. Protoc.* 5 725–738. 10.1038/nprot.2010.5 20360767 PMC2849174

[B81] Salleh F. M. Evans K. Goodall B. Machin H. Mowla S. B. Mur L. A. J. (2012). A novel function for a redox-related LEA protein (SAG21/AtLEA5) in root development and biotic stress responses. *Plant Cell Environ.* 35 418–429. 10.1111/j.1365-3040.2011.02394.x 21736589

[B82] Schaefer C. Schlessinger A. Rost B. (2010). Protein secondary structure appears to be robust under in silico evolution while protein disorder appears not to be. *Bioinformatics* 26 625–631. 10.1093/bioinformatics/btq012 20081223 PMC2828120

[B83] Schmid M. Davison T. S. Henz S. R. Pape U. J. Demar M. Vingron M. (2005). A gene expression map of *Arabidopsis thaliana* development. *Nat. Genet.* 37 501–506. 10.1038/ng1543 15806101

[B84] Schranz M. E. Lysak M. A. Mitchell-Olds T. (2006). The ABC’s of comparative genomics in the Brassicaceae: building blocks of crucifer genomes. *Trends Plant Sci.* 11 535–542. 10.1016/j.tplants.2006.09.002 17029932

[B85] Seo P. J. Kim M. J. Ryu J.-Y. Jeong E.-Y. Park C.-M. (2011). Two splice variants of the IDD14 transcription factor competitively form nonfunctional heterodimers which may regulate starch metabolism. *Nat. Commun.* 2:303 10.1038/ncomms1303 21556057

[B86] Shinozaki K. Yamaguchi-Shinozaki K. Seki M. (2003). Regulatory network of gene expression in the drought and cold stress responses. *Curr. Opin. Plant Biol.* 6 410–417. 10.1016/S1369-5266(03)00092-X 12972040

[B87] Staswick P. E. Su W. Howell S. H. (1992). Methyl jasmonate inhibition of root growth and induction of a leaf protein are decreased in an *Arabidopsis thaliana* mutant. *Proc. Natl. Acad. Sci. U.S.A.* 89 6837–6840. 10.1073/pnas.89.15.6837 11607311 PMC49599

[B88] Stettler M. Eicke S. Mettler T. Messerli G. Hörtensteiner S. Zeeman S. C. (2009). Blocking the metabolism of starch breakdown products in *Arabidopsis* leaves triggers chloroplast degradation. *Mol. Plant* 2 1233–1246. 10.1093/mp/ssp093 19946617 PMC2782796

[B89] Storozhenko S. De Pauw P. Van Montagu M. Inzé D. Kushnir S. (1998). The heat-shock element is a functional component of the *Arabidopsis* APX1 gene promoter. *Plant Physiol.* 118 1005–1014. 10.1104/pp.118.3.1005 9808745 PMC34773

[B90] Sucaet Y. Wang Y. Li J. Wurtele E. S. (2012). MetNet Online: a novel integrated resource for plant systems biology. *BMC Bioinformatics* 13:267 10.1186/1471-2105-13-267 PMC3483157 23066841

[B91] Thieme C. J. Rojas-Triana M. Stecyk E. Schudoma C. Zhang W. Yang L. (2015). Endogenous *Arabidopsis* messenger RNAs transported to distant tissues. *Nat. Plants* 1:15025 10.1038/nplants.2015.25 27247031

[B92] Thompson J. E. Froese C. D. Madey E. Smith M. D. Hong Y. (1998). Lipid metabolism during plant senescence. *Prog. Lipid Res.* 37 119–141. 10.1016/S0163-7827(98)00006-X 9829123

[B93] van der Graaff E. Schwacke R. Schneider A. Desimone M. Flugge U. I. Kunze R. (2006). Transcription analysis of *Arabidopsis* membrane transporters and hormone pathways during developmental and induced leaf senescence. *Plant Physiol.* 141 776–792. 10.1104/pp.106.079293 16603661 PMC1475451

[B94] Weaver L. M. Amasino R. M. (2001). Senescence is induced in individually darkened *Arabidopsis* leaves but inhibited in whole darkened plants. *Plant Physiol.* 127 876–886. 10.1104/pp.010312 11706170 PMC129259

[B95] Windsor A. J. Schranz M. E. Formanová N. Gebauer-Jung S. Bishop J. G. Schnabelrauch D. (2006). Partial shotgun sequencing of the *Boechera stricta* genome reveals extensive microsynteny and promoter conservation with *Arabidopsis*. *Plant Physiol.* 140 1169–1182. 10.1104/pp.105.073981 16607030 PMC1435815

[B96] Wu S. J. Ding L. Zhu J. K. (1996). SOS1 a genetic locus essential for salt tolerance and potassium acquisition. *Plant Cell* 8 617–627. 10.1105/tpc.8.4.617 12239394 PMC161124

[B97] Wu Y. Sanchez J. P. Lopez-Molina L. Himmelbach A. Grill E. Chua N. H. (2003). The abi1-1 mutation blocks ABA signaling downstream of cADPR action. *Plant J.* 34 307–315. 10.1046/j.1365-313X.2003.01721.x 12713537

[B98] Wurtele E. S. Wang H. Durgerian S. Nikolau B. J. Ulrich T. H. (1993). Characterization of a gene that is expressed early in somatic embryogenesis of *Daucus carota*. *Plant Physiol.* 102 303–312. 10.1104/pp.102.1.303 8108498 PMC158776

[B99] Yamamoto Y. Y. Obokata J. (2008). ppdb: a plant promoter database. *Nucleic Acids Res.* 36 D977–D981. 10.1093/nar/gkm785 17947329 PMC2238996

[B100] Yang R. Jarvis D. J. Chen H. Beilstein M. Grimwood J. Jenkins J. (2013). The reference genome of the halophytic plant *Eutrema salsugineum*. *Front. Plant Sci.* 4:46 10.3389/fpls.2013.00046 PMC3604812 23518688

[B101] Yao Y. You J. Ou Y. Ma J. Wu X. Xu G. (2015). Ultraviolet-B protection of ascorbate and tocopherol in plants related with their function on the stability on carotenoid and phenylpropanoid compounds. *Plant Physiol. Biochem.* 90 23–31. 10.1016/j.plaphy.2015.02.021 25749732

[B102] Zimmermann P. Heinlein C. Orendi G. Zentgraf U. (2006). Senescence-specific regulation of catalases in *Arabidopsis thaliana* (L.) Heynh. *Plant Cell Environ.* 29 1049–1060. 10.1111/j.1365-3040.2005.01459.x 17080932

