# Supplementary material for: A Clade-Specific Arabidopsis Gene Connects Primary Metabolism and Senescence
Source: Front Plant Sci. 2016 Jul 12;7:983. doi: 10.3389/fpls.2016.00983 (PMC4940393; doi:10.3389/fpls.2016.00983)
Supplement: Supplementary file 1 [file Data_Sheet_1.PDF]

**Supplementary Material** for

# A clade-specific Arabidopsis gene connects primary metabolism and senescence

Dallas C. Jones[1], Wenguang Zheng[1], Sheng Huang[1†], Chuanlong Du[2], Xuefeng Zhao[3†], Ragothaman M. Yennamalli[1,4†], Taner Z. Sen[1,4], Dan Nettleton[2], Eve Syrkin Wurtele[1,5], Ling Li[1,5]*

[1] Department of Genetics, Development and Cell Biology, Iowa State University, Ames, Iowa, 50011, USA, [†] Present address: State Key Laboratory for Conservation and Utilization of Subtropical Agro-Bioresources, College of Life Science and Technology, Guangxi University, Nanning, Guangxi, 530005, P. R. China, [2] Department of Statistics, Iowa State University, Ames, Iowa, 50011, USA, [3] Laurence H. Baker Center for Bioinformatics and Biological Statistics, Iowa State University, Ames, IA 50011, [†] Present address: Information Technology, College of Liberal Arts and Sciences, Iowa State University, Ames, Iowa, 50011, USA, [4] United States Department of Agriculture-Agriculture Research Service Corn Insects and Crop Genetics Research Unit, Ames, Iowa, 50011, USA, [†] Present address: Department of Biotechnology and Bioinformatics, Jaypee University of Information Technology, Waknaghat, Himachal Pradesh, India 173234, [5] Center for Metabolic Biology, Iowa State University, Ames, Iowa, 50011, USA

***Correspondence:** Ling Li, Department of Genetics, Development and Cell Biology, Iowa State University, Ames, Iowa, 50011, USA, **liling@iastate.edu**

## Supplementary figures

### A Gene alignments (Kalign)

Yellow highlight: Bases conserved among all homologs
Red text: Upstream *Capsella* gene sequence
Green Text: *Arabidopsis thaliana* 5' UTR
Blue text: *Arabidopsis thaliana* CDS
Orange text: *Arabidopsis thaliana* 3' UTR
**Violet text**: *Arabidopsis thaliana* intron sequence
Blue highlight: CDS start sites
Green highlight: Intron start sites

```
A. thaliana       1 CATCCACTTGAAATGAATCTAATTCATAAAATGATGTAACAGAAGCTTGAGGAATCCAAA
A. lyrata         1 CATCCACTTGAAATGAATCTAATTCATAAAATGATGTAACAAAAGCTTGAGGAATCCAAA
B. stricta        1 CATCCACTTGAAATGAATCTAATTCATAGTTTGATGTAACAAAAGTTTGAGAAATCCAAA
C. grandiflora    1 CATCCACTTGAAATGAATCTAATTCATAATTTGATGTAACAAAAGTTTGAGGAATC-AAA
C. rubella        1 CATCCACTTGAAATGAATCTAATTCATAATTTGATGTAACAAAAGTTTGAGGAATC-AAA
consensus         1 CATCCACTTGAAATGAATCTAATTCATAattTGATGTAACAaAAGtTTGAGgAATCcAAA

A. thaliana      61 CAATGTACATAC---------GTTAATACAACATGCAAGTTCATCTATAAATATGGAACC
A. lyrata        61 CAATGTGCAAACTTATGATAAGTTCATACAACATGCAACTTCATCTATAAATATGGAACC
B. stricta       61 CAATGTGCATAC---------GTACATACAACATACAAATTCATCTATAAATATAG----
C. grandiflora   60 CAATGTGCATAC---------GTACATTACA-----AATTTCATCTATAAATATTG----
C. rubella       60 CAATGTGCATAC---------GTACATTACA-----AATTTCATCTATAAATATTG----
consensus        61 CAATGTgCAtAC         GTacATacaAcatgcAA TTCATCTATAAATATgG

A. thaliana     112 TTAACTCTCGGATAAGAATTCACCAAGGCAATACATTTT-----ACCTAAG-------AG
A. lyrata       121 TTAACTCTC------GAATTCACCAAGGCAATACATTTT-----AGCTAAACAAAAAAAT
B. stricta      108 -------------------TAACCTAGGCAATACATTTTCGAAACGATAAAGAAGAAGAA
C. grandiflora  102 -------------------TACCTAGGCAAATACATTTT-----AGATATGATAAAATAA
C. rubella      102 -------------------TACCTAGGCAAATACATTTT-----AGATATGATAAAATAA
consensus       121                    TaaCcaaGgcAATACATTTT     agaTAagg aaaa Aa

A. thaliana     160 AACAGAA-------AAAAGACACTTAGCAGGTGAAACAATGTCGTTTAGAAAAGTAGAGA
A. lyrata       170 AAGAGAA-------AAACAAAACTTTGTAGGTGAATCGATGTCGTTTAGAAAAGTAGAGA
B. stricta      149 AAAAAAA-------AAAAGACACTTGGCAGGGGAAATGATGTCGTTTAGAAACGTAGAGA
C. grandiflora  138 AGAAGAGGATAATCAAAAGACACATAGCGGGTGAAACGATGTCGTTTAGGAAAGTGGAGA
C. rubella      138 AGAAGAGGATAATCAAAAGACACATAGCGGGTGAAACGATGTCGTTTAGGAAAGTGGAGA
consensus       181 AaaAgAa       AAAagAcACtTaGcaGGtGAAacgATGTCGTTTAGaAAaGTaGAGA

A. thaliana     213 AGAAACCAACTGAAATGGGCCGAAACATGACCCACGAGAAGTCGGACTCTGATTCGGACA
A. lyrata       223 AGAAACCAACCGAAATGGGCCGTCAAATGACACACGAGAAGTCGGACTCTGACTCAGACA
B. stricta      202 AGAAATCAACCGAAATGAGCCGACACATGACCCACGAGAAGTCGGAATCTGATTCGGACA
C. grandiflora  198 AGAAACCAACCGAAATGGGCCGACACATGACCCACGAGAAGTCGAACTCTGATTCGTACA
C. rubella      198 AGAAACCAACCGAAATGGGCCGACACATGACCCACGAGAAGTCGAACTCTGATTCGTACA
consensus       241 AGAAAcCAACcGAAATGgGCCGacAcATGACcCACGAGAAGTCGgAcTCTGAtTCggACA

A. thaliana     273 ACGAAGGAGCCCCCATGACTGTTGGAGGTTACACTGAATTCGTTGCCCGCAGCGAC---T
A. lyrata       283 ACGAAGGAACCCCCATGACGGCTGGCGGTTACACTGAATTCGTTGCCCGCAGCGAC---T
B. stricta      262 ACGAAAGAACCCCCATGATGGCTCCAGGTTACAGTGAATATGTTACACGCAGCGAC---T
C. grandiflora  258 AAGAAGGAGCTCCCATGATGGCTCCAGGTTACACTGAAATGGTTAGCCGCAGCGACGAGT
C. rubella      258 AAGAAGGAGCTCCCATGATGGCTCCAGGTTACACTGAAATGGTTAGCCGCAGCGACGAGT
consensus       301 AcGAAgGAgCcCCCATGAtgGcTccaGGTTACAcTGAAtt GTTaccCGCAGCGAC   T
```

```
A. thaliana     330 CTGACTGGGACGAACCGGTTTATTCTGGCAAAGCCCGTTCTAATTATAACCTCACCGGCA
A. lyrata       340 CAGACTGGGACGAACCTTTTTATTCTGGCAAAGCCCGTTCTAA------------CCTCA
B. stricta      319 CTGACTGGGACGAACCGGTTGATTCTCGCAAACGCAGTCCTGA------------CCCCA
C. grandiflora  318 CTGACTGGGACGATCTTGTTGACTCTGGCAAAACCCGTGCTAG------------CCTCA
C. rubella      318 CTGACTGGGACGATCTTGTTGACTCTGGCAAAACCCGTGCTAG------------CCTCA
consensus       361 CtGACTGGGACGAaCctgTTgAtTCTgGCAAAgcCcGT CTaa            CctCA

A. thaliana     390 CCGCAAAAGGAACTGGTCCCATCAATAGCTTCAGCCGCAAACACTTCCCTAATTACTAAT
A. lyrata       388 CCACCAAGGAAACTGGTCCCACCAATAGCTACAGCCGCAAACATTTCTCTAATAACTGAT
B. stricta      367 TTGCCAAGGAAACTGGTCCTATCAATCGCTACAGCCGCAAATATTACTCTAATTACTAAG
C. grandiflora  366 CCGCCATGGAAACTGCTCCTATCAATCGTTTCAGCCGCAAGAATTTCTCTAATTACTAAG
C. rubella      366 CCGCCATGGAAACTGCTCCTATCAATCGTTTCAGCCGCAAGAATTTCTCTAATTACTAAG
consensus       421 ccgCcAagGaAACTGgTCCtAtCAATcGcTtCAGCCGCAAa AtTtCtCTAATtACTaAg

A. thaliana     450 CATGTA------AGCGGTTTGAACTCTATAATCAAGATTTAAAAGACGCTTTAAATTCGT
A. lyrata       448 CATGTA------AGAGGTTCGAATTCTATAATCAAGATT---AAGATTCTTGAAAATCAT
B. stricta      427 CATGTA------AGAGGTTCGAGTTCTAAAATCAAGATT---TAGATACTTGAAAATCAT
C. grandiflora  426 CATGCATGTAAGAGAGGTTCGAA-------------------------------------
C. rubella      426 CATGCATGTAAGAGAGGTTCGAA-------------------------------------
consensus       481 CATGtA      AGaGGTTcGAa tcta aatcaagatt    aga  ctt aaa tcgt

A. thaliana     504 AGAT-ATATCAATTATTGT-----------TAATTTTACATATTTTGCGTTATGTTTTT-
A. lyrata       499 GAATTACGTCATTTATTTT-----------------------TTTTCCGTTATGTTTTTT
B. stricta      478 GAAT-ATGTAACTAATTGTATGGTACCTTTTAATTTTATTTGTTTTGGGTTATGTTTT--
C. grandiflora  449 -----TTGTAGTTTATTGTATGGTTCCCTTTAATTTTATTTGTTTTGCGTTATGATTTT-
C. rubella      449 -----TTGTAGTTTATTGTATGGTTCCCTTTAATTTTATTTGTTTTGCGTTATGATTTT-
consensus       541 agat atgTaatTtATTgTatggt cc tttaattttatttgTTTTgcGTTATGtTTTt

A. thaliana     551 CAGATGGGCAGTGCTACGTAAAAGTGAGAAA-CGAAGACATGCAGGCTCACTAGCTAGTT
A. lyrata       536 CAGATGGGCTTGGCAACGTAAAAGTGAGAAA-TGAAGATATGCAAGCTCATTA----GTT
B. stricta      535 CAGATGGGCTTGGCTACGTAAAAGGGAAAAAATGAAGACATGCTCATTAGTTA-------
C. grandiflora  503 CAGATG-GCTTGACAACGTAAAAGTGAAAAA-TGAAGACATGCTCATTAGGTG-------
C. rubella      503 CAGATG-GCTTGACAACGTAAAAGTGAAAAA-TGAAGACATGCTCATTAGGTG-------
consensus       601 CAGATGgGCttggCaACGTAAAAGtGAaAAA tGAAGAcATGCtcatTag Ta

A. thaliana     610 GAATAATGCCCATATAAATT---ATGTACTATGTCTGAATGTAATAAATAAGAAT-----
A. lyrata       591 AAATAATGCCCATATAACTT---ATGTATTATGTCTGAATGTCATAAATAAGAAT-----
B. stricta      588 ----AAGACTCATATAAATT---ATGTATTATGTCTGAATGTAATAAATAAGAAT-----
C. grandiflora  554 ----AAGGCTCATATGAATTAGTATGTATTATGTCTGAATGTAATGAATAAGAATATAAG
C. rubella      554 ----AAGGCTCATATGAATTAGTATGTATTATGTCTGAATGTAATGAATAAGAATATAAG
consensus       661     AAggCtCATATaAaTT   ATGTAtTATGTCTGAATGTaATaAATAAGAAT

A. thaliana     662 -------CCAAGGATGGATGATGACGCATGAAC----ATATCAATGATTATAGTCTCATT
A. lyrata       643 -------CCAAGGATGGATGATGCCGCATGAAC----ATATCAATGATTATAGTCTCATT
B. stricta      636 -------CCAAGGATGGAT---GCCTCATGAAA----ATATCAGTGATGATAATCTCATT
C. grandiflora  610 AATTAATCCAAGGATGATGGATGCCTCATGAACGAACATATCGGTGATGACAATCTCATT
C. rubella      610 AATTAATCCAAGGATGATGGATGCCTCATGAACGAACATATCGGTGATGACAATCTCATT
consensus       721        CCAAGGATGgatgatGcCtCATGAAc    ATATCagTGATgAtAaTCTCATT

A. thaliana     711 GTGCTTTCATGCAGTATTTATACAAAGCACTTATGGAAT-----------GAGTATGTTA
A. lyrata       692 GG--TTTCATGCGGT-TTTATACAAAGCAACTATGCAACTATGGAATGAGAAGAATGTTA
B. stricta      682 GG--TATCATGCGGTTTTTATGCAAAGCAACTATAGAAT-----------AAGAATGTTA
C. grandiflora  670 GG--TATTATGCGGTTTATATACAAAGTAACTATGGAAT-----------GATAATGTTA
C. rubella      670 GG--TATTATGCGGTTTATATACAAAGTAACTATGGAAT-----------GATAATGTTA
consensus       781 Gg  TaTcATGCgGTtTtTATaCAAAGcAacTATggAAt           gAgaATGTTA
```

```
A. thaliana      760  CATAACTCTACCTATACTTCTATTATTATGAACATATTAATTATAT--------------
A. lyrata        749  CATAACTCTAACTACTTCTATATTATTGTGAACAAATTAGTT---T--------------
B. stricta       729  TAT-ATAATATGGCTACTTCTAATATTATGAACAAGTAAATAGACC--------------
C. grandiflora   717  CTTAATTATAGCTACTTCTATTATATTATGAACAAGTAAATTATATCAATATGCCAAATT
C. rubella       717  CTTAATTATAGCTACTTCTATTATATTATGAACAAGTAAATTATATCAATATGCCAAATT
consensus        841  caTaAttaTAactacttcTaTaaTATTaTGAACAagTaAaTtatat

A. thaliana      806  ---------ATA----------------AAAAAAAGCTTAATCTTTTACAATACTGTAAG
A. lyrata        792  ---------AAA----------------AATAAAAGCAGAATAATTTACAATACTATAAA
B. stricta       774  ---------AAATTAATTTCAAAACTTCAAATCTAGAATAATATTTTACAATACTTTAAA
C. grandiflora   777  CGTTTCGTCAAA--AAGTTTA-------AAATCTAGAATAATTTTTTACAAGACTATGTA
C. rubella       777  CGTTTCGTCAAA--AAGTTTA-------AAATCTAGAATGATTTTTTACAAGACTATGTA
consensus        901           AaA  aa tt a       AAatctAGaataAT tTTTACAAtACTaTaaa

A. thaliana      841  AGATGAGAACAATTGTAACAATTCCACACCACACCAAAACAAAAATAAAAAAATTGTAAC
A. lyrata        827  AGATGAGAACAGT------------------------------------------GTAAC
B. stricta       825  GGCC-------------------------------------------------TTGTAAT
C. grandiflora   828  ACAT------------------TCC------------------------------GTAAC
C. rubella       828  ACAT------------------TCC------------------------------GTAAC
consensus        961  agat                  tcc                              GTAAc

A. thaliana      901  AATTCCACATGTTATAGTCTTCTAGATTGCAATGA--TCAATATGAATCAAAGAATCTGA
A. lyrata        845  AATTCCACACGTTATAGTCTTCTAGATTGCAATGA--TCAATATGAATCAAAGAATCTGA
B. stricta       836  AATTCCCCCTA--------TTCTAGATCGCAATGAGATCAATATGAATC-----------
C. grandiflora   840  AATTCTCCTTT-------------------------------------------------
C. rubella       840  AATTCTCCTT--------------------------------------------------
consensus       1021  AATTCccC tg        ttctagat gcaatga  tcaatatgaatc

A. thaliana      959  ATCTTGGACACCTATAATTGGGAACCAATTCTGTAATTGACATCCAAGTTAGGTAAAAAG
A. lyrata        903  ATCTTGGACACCTATAACTGAGAACCAATTCTGAGATTGACATCCAAA-TAGGTAAAAAG
B. stricta            ------------------------------------------------------------
C. grandiflora        ------------------------------------------------------------
C. rubella            ------------------------------------------------------------
consensus       1081
A. thaliana     1019  AAAATGCCATTCGAATTGATTCAATTTT-------------
A. lyrata        962  AAAATGCCATTCGAATTGATTCAATGTTAGCTAACTTTAGT
B. stricta            -----------------------------------------
C. grandiflora        -----------------------------------------
C. rubella            -----------------------------------------
consensus       1141
```

### *Arabidopsis halleri*

*>A. halleri*

ATGTCGTTTAGAAAAGTAGAGAAGAAACCAACCGAAATGGGCCGACAGATGACCCACGAGAAGTCGGACTCTGACTC
AGACAACGAAGGAACCCCCATGACGGCTGGCG

```
A. thaliana    1  ATGTCGTTTAGAAAAGTAGAGAAGAAACCAACTGAAATGGGCCGAAACATGACCCACGAG
A. halleri     1  ATGTCGTTTAGAAAAGTAGAGAAGAAACCAACCGAAATGGGCCGACAGATGACCCACGAG
A. lyrata      1  ATGTCGTTTAGAAAAGTAGAGAAGAAACCAACCGAAATGGGCCGTCAAATGACACACGAG
consensus      1  ATGTCGTTTAGAAAAGTAGAGAAGAAACCAACcGAAATGGGCCGacAgATGACcCACGAG

A. thaliana   61  AAGTCGGACTCTGATTCGGACAACGAAGGAGCCCCCATGACTGTTGGAG-
A. halleri    61  AAGTCGGACTCTGACTCAGACAACGAAGGAACCCCCATGACGGCTGGCGG
A. lyrata     61  AAGTCGGACTCTGACTCAGACAACGAAGGAACCCCCATGACGGCTGGCGG
```

```
consensus        61 AAGTCGGACTCTGAcTCaGACAACGAAGGAaCCCCCATGACgGcTGGcGg
```

## B SAQR (*AT1G64360*)

### Promoter sequence

```
aaagcttgatggagaagaaaaggttgaggttcttttgcaataattagcatttaacacatagtaagaactttttttgtgcccat
tatgattgataggggttggcaaaaaaccttaaaacaagctctgatcaaaagagaagaaccttgttgaggccaaaaccatatt
gggaatggtattactatttatcatattatggagaggaatggtagaagaggatcatcaacggctgatactaaagatttttgcc
tagtaaagaaaaagttgcatgaaacttggaactagttcattacacaccataaaaattaatacatatgatacaaggaattcag
aacatttctttggtccaaggatacaaatcacaaaaaaaaaagacatatatttggtagaatgtagtggtcatcacgactttaac
ataaacgacacaaaagaaaaatcgtctttcaagtgaatttttgttgtaacgtatacttatctatactgatagtaacacatat
agtcaaaacacaaaaggtgaagattgtaaatctcatccacttgaaatgaatctaattcataaaatgatgtaacagaagcttg
aggaatccaaacaatgtacatacgttaatacaacatgcaagttcatctataaatatggaaccttaactctcggataagaatt
caccaaggcaatacattttacctaagagaacagaaaaaagacacttagcaggtgaaaca
```

### Protein sequence

```
MSFRKVEKKP TEMGRNMTHE KSDSDSDNEG APMTVGGYTE FVARSDSDWD
EPVYSGKARS NYNLTGTAKG TGPINSFSRK HFPNY
```

**Figure S1. *SAQR* gene information. (A)** *SAQR* homologs are present in *Arabidopsis lyrata*, *A. halleri*, *Capsella rubella*, *C. grandiflora*, and *Bochera stricta*. Genome sequences and gene models of the six *SAQR* homologs downloaded from Phytozome 10.2 (http://phytozome.jgi.doe.gov/pz/portal.html). *SAQR* homologs are conserved within their 5′ UTR and CDS regions and share a similar intron structure. Genome sequences aligned using Kalign (http://www.ebi.ac.uk/Tools/msa/kalign/). **(B)** *SAQR* promoter sequence and protein sequence in *A. thaliana*.

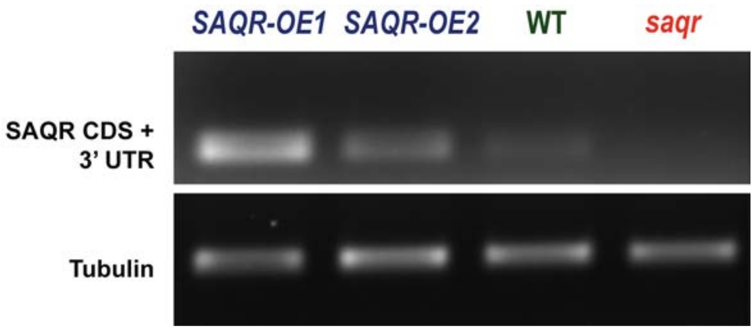


**Figure S2. Semi-quantitative RT-PCR of *SAQR* in extracts from seedling shoots of *SAQR-OE*, wild type, and *saqr* lines.** The approximately 300 bp fragment spans from the beginning of the CDS to a section of the 3′ UTR. PCR using tubulin primers was used as control.

**A**

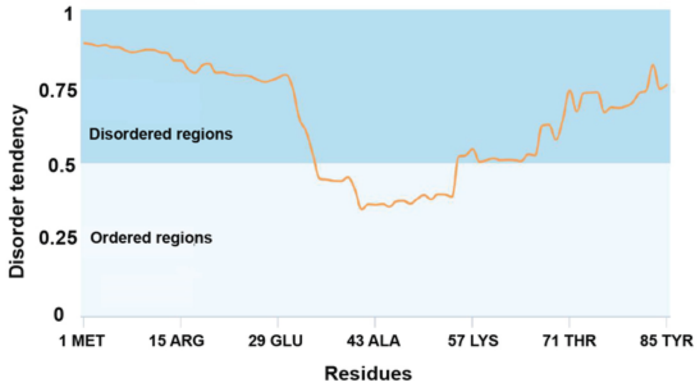


**B**

```
         10         20         30         40         50         60
MSFRKVEKKP TEMGRNMTHE KSDSDSDNEG APMTVGGYTE FVARSDSDWD EPVYSGKARS
         70         80
NYNLTGTAKG TGPINSFSRK HFPNY
```

Sequence Length: 85

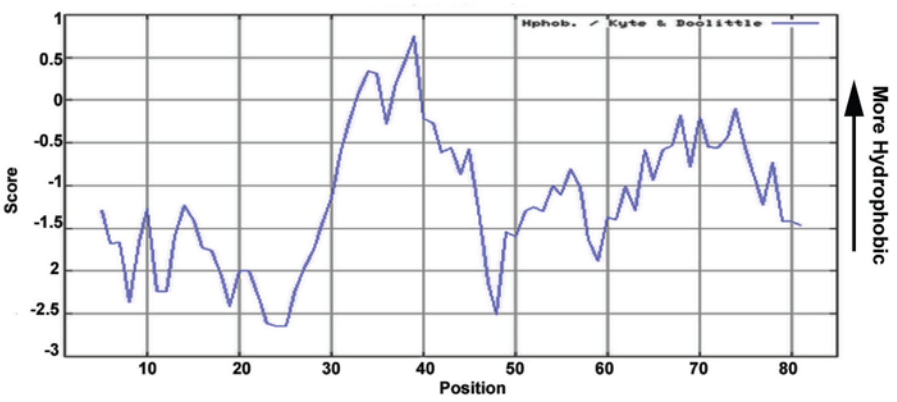


Using the scale of Hydrophobicity from Kyle & Doolittle, 1982, the individal values for the 20 amino acids are:

Ala: 1.800 Arg: -4.500 Asn: -3.500 Asp: -3.500 Cys: 2.500 Gln: -3.500
Glu: -3.500 Gly: -0.400 His: -3.200 Ile: 4.500 Leu: 3.800 Lys: -3.900
Met: 1.900 Phe: 2.800 Pro: -1.600 Ser: -0.800 Thr: -0.700 Trp: -0.900
Tyr: -1.300 Val: 4.200

**Figure S3**. **Predictive analysis of the SAQR protein. (A)** Predicted disorder of the SAQR protein sequence using MetaDisorderMD2 (Kozlowski and Bujnicki, 2012) (http://genesilico.pl/metadisorder/). Global disorder tendency for whole sequence is 0.642. All residues with a disorder probability over 0.5 are considered as disordered. **(B)** Prediction of hydrophilicity used Protscale software (http://web.expasy.org/protscale/) indicates SAQR is largely hydrophilic. Hydrophilicity scale was adapted from (Kyte and Doolittle, 1982), hydrophilicity increases with lower scores.

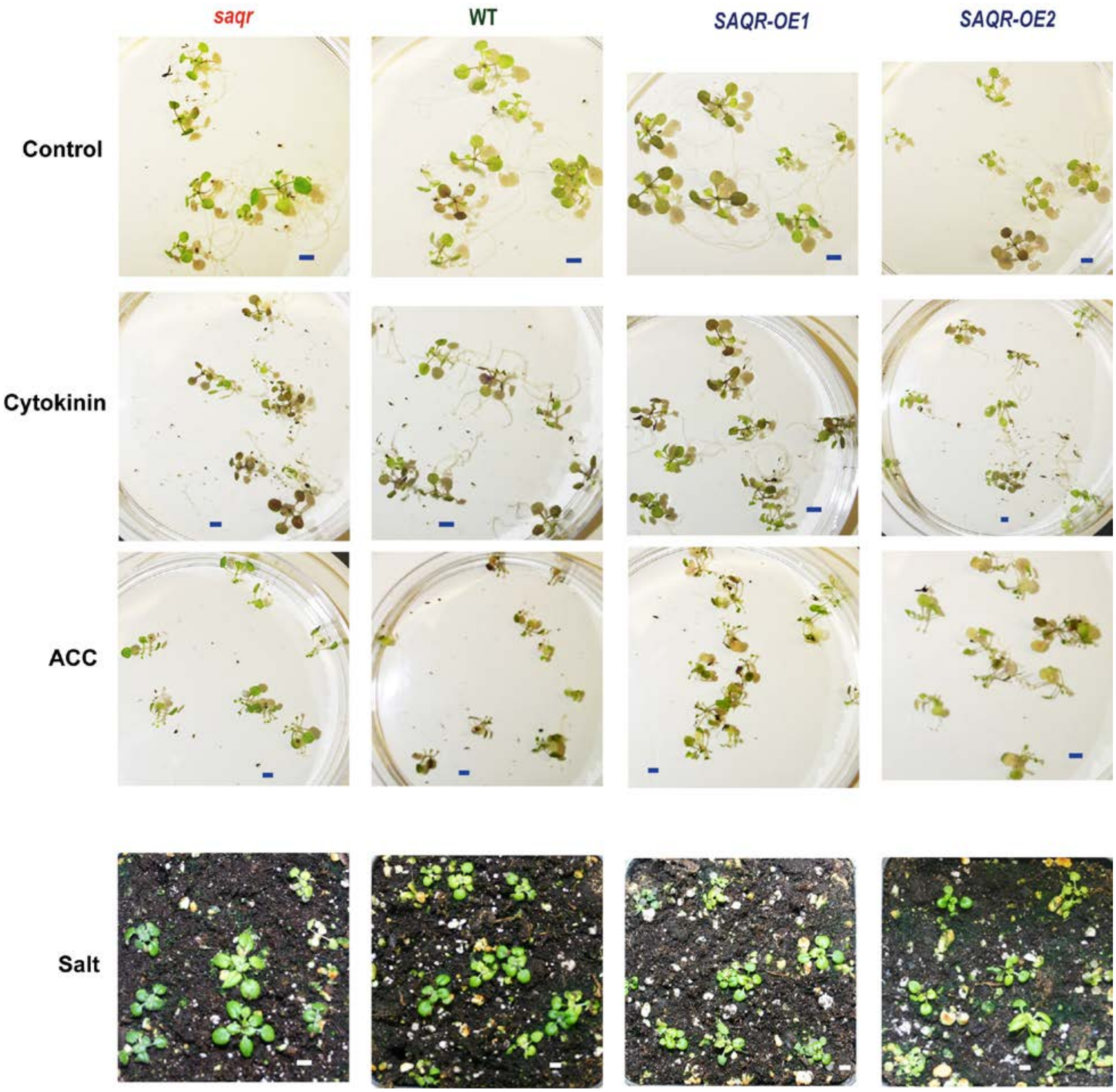


**Figure S4**. **Visible phenotypes of *SAQR* knockout (*saqr*) and overexpression (OE) lines were similar to those of WT controls when plants were treated with cytokinin, ACC, or salt stress.** Twelve-DAI soil-grown seedlings were moved and placed for four days under constant light in water (control), water containing 1 µM kinetin (cytokinin), or 50 µM 1-aminocyclopropane-1-carboxylic acid (ACC; ethylene). Twelve-DAI soil-grown seedlings were watered for four days with 200 mM sodium chloride (salt), as treating the plants in soil with salt water mimics a condition a plant could have in nature. Blue bar and white bar, 2 mm.

## Supplementary tables

**Table S1. Cis-acting motifs in the *SAQR* promoter region upstream of the transcription start site (-715 to -58 bp).** Analyses were performed using Athena (O'Connor et al., 2005), Plant Care (http://bioinformatics.psb.ugent.be/webtools/plantcare/html/), and the Plant Promoter Database (Yamamoto and Obokata, 2008). TTS, transcription start site.

| Name of Motif | Annotation | Position Start | Position End (from TSS) | Sequence | Source |
|---|---|---|---|---|---|
| TCA-element | Cis-acting element involved in salicylic acid responsiveness | -646 | -636 | GAGAAGAAAA | PlantCARE |
| CARGCW8GAT | Binding site for AGL15 | -620 | -611 | CAATAATTAG | Athena |
| CAAT-box | Common cis-acting element in promoter and enhancer regions | -620 | -616 | CAAT | PlantCARE |
| G-box | Cis-acting regulatory element involved in light responsiveness | -606 | -596 | TAACACATAG | PlantCARE |
| MNF1 | Light responsive element | -583 | -567 | GTGCCCATTATGATTA | PlantCARE |
| GATA-motif | Part of a light responsive element | -568 | -561 | GATAGGG | PlantCARE |
| LAMP-element | Part of a light responsive element | -506 | -497 | CCAAAACCA | PlantCARE |
| MYB1AT | Stress response, ABA signaling | -503 | -498 | AAACCA | Athena |
| CCAAT-box | MYBHv1 binding site | -438 | -432 | CAACGG | PlantCARE |
| AAGAA-motif (A. Satvia) | N/A | -410 | -400 | AGTAAAGAAA | PlantCARE |
| Box 4 | Part of a conserved DNA module involved in light responsiveness | -357 | -351 | ATTAAT | PlantCARE |
| ATGCAAAT motif | Cis-acting regulatory element associated to the TGAGTCA motif | -309 | -301 | ATACAAAT | PlantCARE |
| CAAT-box | Common cis-acting element in promoter and enhancer regions | -306 | -301 | CAAAT | PlantCARE |
| CARGCW8GAT | Binding site for AGL15 | -287 | -278 | CATATATTTG | Athena |
| Skn-1_motif | Cis-acting regulatory element required for endosperm expression (Oryza sativa) | -264 | -259 | GTCAT | PlantCARE |
| G-box | Cis-acting regulatory element involved in light responsiveness | -259 | -253 | CACGAC | PlantCARE |
| ATREG527 | DREB1Aox (overexpression of DREB1A) | -248 | -237 | TAAACGAC | PPDB |
| ATREG569 | | -247 | -236 | AAACGACA | PPDB |
| ATREG565 | | -246 | -239 | TAAACGAC | PPDB |
| HSE | Cis-acting element involved in heat stress responsiveness | -233 | -224 | AGAAAATTCG | PlantCARE |
| ATCT-motif | Part of a conserved DNA module involved in light responsiveness | -137 | -127 | AATCTCATCC | PlantCARE |
| ATCT-motif | Part of a conserved DNA module involved in light responsiveness | -117 | -107 | AATCTAATTC | PlantCARE |
| CAAT-box | Common cis-acting element in promoter and enhancer regions | -72 | -68 | CAAT | PlantCARE |
| Circadian | Cis-acting regulatory element involved in circadian control | -46 | -36 | CAAGTTCATC | PlantCARE |
| TATA-box Motif | Core promoter element around -30 of transcription start | -36 | -31 | TATAAAA | Athena |

| | | | | | |
|---|---|---|---|---|---|
| Ibox promoter motif | Part of a light responsive element | -11 | -6 | GATAAG | Athena |

Red=stress responsive/regulatory
Yellow=light response/circadian
Blue=flowering/growth

**Table S2. Secondary structure composition for each predicted protein model calculated using Promotif.** Number of residues (out of 85) and the percentages are given for each predicted protein model (Hutchinson and Thornton, 1996). Refer to **Figure 1B** for model illustrations.

| At1g64360 | β-strand - number of residues (percentage) | α-helix - number of residues (percentage) | 3-10 helix - number of residues (percentage) | Loop - number of residues (percentage) |
|---|---|---|---|---|
| **Model 1** | 4 (4.7%) | 9 (10.6%) | 3 (3.5%) | 69 (81.2%) |
| **Model 2** | 0 (0.0%) | 9 (10.6%) | 6 (7.1%) | 70 (82.4%) |
| **Model 3** | 0 (0.0%) | 11 (12.9%) | 0 (0.0%) | 74 (87.1%) |
| **Model 4** | 0 (0.0%) | 4 (4.7%) | 3 (3.5%) | 78 (91.8%) |
| **Model 5** | 9 (10.6%) | 9 (10.6%) | 0 (0.0%) | 67 (78.8%) |

**Table S3. Genes positively correlated to the *SAQR* transcript profile.** Genes positively correlated by Spearman's correlation (≥0.7). Pathway and regulon data are derived from AtGeneSearch (http://www.metnetdb.org/MetNet_atGeneSearch.htm) (Mentzen and Wurtele, 2008; Sucaet et al., 2012). mRNA transcriptome profiling dataset ("At956-2008", from 956 Affymetrix ATH1 chips) was used (Li et al., 2007; Mentzen and Wurtele, 2008; Li et al., 2009).

| Correlation | Locus ID | Gene | Pathways | Regulons | Probe Name |
|---|---|---|---|---|---|
| 1.00 | AT1G64360 | SAQR | | | 259766_at |
| 0.86 | AT3G15353 | MT3; METALLOTHIONEIN 3 | | 57 - phloem specific (vasculature tissues - specific) | 257054_at |
| 0.84 | AT5G10380 | | | 35 - kinases, signaling, disease resistance | 250435_at |
| 0.83 | AT4G00780 | | | 57 - phloem specific (vasculature tissues - specific) | 255626_at |
| 0.82 | AT3G61210 | EMB34 | | 57 - phloem specific (vasculature tissues - specific) | 251360_at |
| 0.82 | AT5G11060 | KNAT4 | | 139 | 245901_at |
| 0.82 | AT1G23130 | | | 57 - phloem specific (vasculature tissues - specific) | 264899_at |
| 0.81 | AT1G67860 | | | 57 - phloem specific (vasculature tissues - specific) | 260004_at |
| 0.81 | AT3G53960 | | | | 251916_at |
| 0.81 | AT3G04210 | | | 25 - defense response | 258537_at |
| 0.79 | AT2G14560 | | | 25 - defense response | 265837_at |
| 0.79 | AT2G32880; AT2G32870 | | | 57 - phloem specific (vasculature tissues - specific) | 267644_s_at |
| 0.79 | AT1G67865 | | | 57 - phloem specific (vasculature | 260012_at |

| | | | | | |
|---|---|---|---|---|---|
| | | | | tissues - specific) | |
| 0.79 | AT2G17040 | ANAC036 | | 35 - kinases, signaling, disease resistance | 263584_at |
| 0.79 | AT2G03530 | UPS2 | | 232 | 265713_at |
| 0.79 | AT5G41410 | BEL1; BELL 1 | | 139 | 249309_at |
| 0.79 | AT1G64370 | | | 57 - phloem specific (vasculature tissues - specific) | 259765_at |
| 0.78 | AT4G19840 | ATPP2-A1 | | 57 - phloem specific (vasculature tissues - specific) | 254551_at |
| 0.78 | AT1G65800; AT1G65790 | | | 25 - defense response | 262926_s_at |
| 0.77 | AT3G28080 | | | 2 - photosynthesis | 257300_at |
| 0.77 | AT1G31170 | | | 464 | 263703_at |
| 0.77 | AT3G04890 | | | 2 - photosynthesis | 259091_at |
| 0.77 | AT3G14620 | CYP72A8; CYTOCHROME P450 | | 25 - defense response | 258063_at |
| 0.76 | AT5G39030 | kinase | | 232 | 249486_at |
| 0.76 | AT1G72910; AT1G72930 | | | 25 - defense response | 262374_s_at |
| 0.76 | AT1G21270 | WAK2 | | 25 - defense response | 259560_at |
| 0.76 | AT5G43440 | 1-AMINOCYCLOPROPANE-1-CARBOXYLATE OXIDASE; oxidoreductase, acting on paired donors, with incorporation or reduction of molecular oxygen, 2-oxoglutarate as one donor, and incorporation of one atom each of oxygen into both donors | ethylene biosynthesis from methionine | 537 | 249128_at |
| 0.76 | AT3G26170; AT3G26180 | | | 232 | 257634_s_at |
| 0.76 | AT1G13090 | CYP71B28; CYTOCHROME P450 MONOOXYGENASE | | 139 | 262780_at |

| | | | | | |
|---|---|---|---|---|---|
| 0.75 | AT3G15840 | | | 88 | 258223_at |
| 0.75 | AT4G23260 | | | 25 - defense response | 254247_at |
| 0.75 | AT1G06460 | ACD32.1; ACD31.2; ALPHA CRYSTALLIN | | 88 | 262629_at |
| 0.75 | AT4G37560 | FORMAMIDASE | | 2 - photosynthesis | 253048_at |
| 0.75 | AT5G44870 | | | 25 - defense response | 249029_at |
| 0.75 | AT1G29395 | COR414-TM1 | | 85 | 259789_at |
| 0.75 | AT5G35480 | | | 2 - photosynthesis | 249726_at |
| 0.75 | AT4G10000 | | | 2 - photosynthesis | 255013_at |
| 0.75 | AT1G68600 | | | 88 | 262232_at |
| 0.74 | AT2G32290 | AtBAM2; BAM2; beta amylase; BETA-AMYLASE | starch and cellulose biosynthesis; starch degradation | 393 | 266357_at |
| 0.74 | AT2G29350 | SAG13; TROPINONE REDUCTASE; alcohol dehydrogenase | | 607 | 266292_at |
| 0.74 | AT5G50160 | oxidoreductase | | 888 | 248540_at |
| 0.74 | AT5G13720 | | | 559 | 250247_at |
| 0.74 | AT5G02790 | IN2 ZEA; catalytic | | 2 - photosynthesis | 250967_at |
| 0.74 | AT5G54610 | | | 25 - defense response | 248169_at |
| 0.74 | AT4G27800 | PHOSPHATASE PPH1 | | 2 - photosynthesis | 253834_at |
| 0.74 | AT5G10760 | | | 35 - kinases, signaling, disease resistance | 250445_at |
| 0.74 | AT3G56710 | SIB1; SIGMA FACTOR BINDING PROTEIN 1 | | 35 - kinases, signaling, disease resistance | 246293_at |

| | | | | | |
|---|---|---|---|---|---|
| 0.74 | AT4G13810 | | | 25 - defense response | 254735_at |
| 0.74 | AT2G29120 | ATGLR2.7; GLR2.7 | | 35 - kinases, signaling, disease resistance | 266782_at |
| 0.74 | AT4G03410 | | | 612 | 255429_at |
| 0.74 | AT2G15580 | | | 837 | 265472_at |
| 0.73 | AT3G45860 | | | 25 - defense response | 252549_at |
| 0.73 | AT3G58010 | | | 2 - photosynthe sis | 251559_at |
| 0.73 | AT1G07280 | | | 88 | 261075_at |
| 0.73 | AT2G47880 | GLUTAREDOXIN; arsenate reductase (glutaredoxin) | | | 266516_at |
| 0.73 | AT1G52870 | | | 2 - photosynthe sis | 260155_at |
| 0.73 | AT3G11660 | NHL1 | | 491 | 259102_at |
| 0.73 | AT1G66920; AT1G66910 | | | 10 - protein modification , defense response | 245760_s _at |
| 0.73 | AT1G78200 | PHOSPHATASE 2C; PHOSPHATASE-2C | | 464 | 260057_at |
| 0.73 | AT5G11840 | | | 139 | 250340_at |
| 0.73 | AT1G75410 | BLH3; BEL1-like homeodomain 3 | | 231 | 261120_at |
| 0.73 | AT2G37540 | OXIDOREDUCTASE; protochlorophyllide reductase | chlorophyll biosynthesis | 25 - defense response | 267169_at |
| 0.73 | AT1G31940 | | | 464 | 255721_at |
| 0.73 | AT5G52750 | | | 35 - kinases, signaling, disease resistance | 248327_at |
| 0.73 | AT4G13140 | | | 2 - photosynthe sis | 254750_at |
| 0.73 | AT2G03310 | | | 127 | 266707_at |
| 0.73 | AT5G38980 | | | 2 - photosynthe sis | 249482_at |
| 0.73 | AT4G04570; AT4G04540 | | | 25 - defense response | 255344_s _at |

| | | | | | |
|---|---|---|---|---|---|
| 0.73 | AT3G57260 | BGL2; BETA-1,3-GLUCANASE; BETA-1,3-GLUCANASE 2; BG2; BG2 PR-2; PATHOGENESIS-RELATED PROTEIN 2; PR2; glucan 1,3-beta-glucosidase | cellulose biosynthesis | 35 - kinases, signaling, disease resistance | 251625_at |
| 0.73 | AT4G23610 | | | 35 - kinases, signaling, disease resistance | 254229_at |
| 0.73 | AT5G61520 | MONOSACCHARIDE STP3 | | | 247529_at |
| 0.73 | AT5G38990; AT5G39000 | | | 25 - defense response | 249480_s_at |
| 0.73 | AT4G14400 | ACD6; ACCELERATED CELL DEATH 6 | | 25 - defense response | 245265_at |
| 0.73 | AT5G14970 | | | 2 - photosynthesis | 246547_at |
| 0.73 | AT2G15040 | | | 25 - defense response | 265893_at |
| 0.73 | AT4G23140; AT4G23160 | | | 35 - kinases, signaling, disease resistance | 254265_s_at |
| 0.73 | AT3G08870 | kinase//kinase | | 25 - defense response | 258982_at |
| 0.73 | AT1G71480 | | | 2 - photosynthesis | 259943_at |
| 0.72 | AT3G28540 | ATPase | | 25 - defense response | 256596_at |
| 0.72 | AT1G51805 | | | 25 - defense response | 256168_at |
| 0.72 | AT5G18240 | MYR1; TRANSFACTOR | | 731 | 250031_at |
| 0.72 | AT1G02475 | | | 2 - photosynthesis | 260916_at |
| 0.72 | AT5G27280 | | | 143 | 246791_at |
| 0.72 | AT3G50950 | shikimate kinase | chorismate biosynthesis | 24 - defense response | 252126_at |
| 0.72 | AT3G26230 | CYP71B24; CYTOCHROME P450 | | 25 - defense response | 257625_at |
| 0.72 | AT4G09760 | CHOLINE KINASE | phospholipid biosynthesis; phospholipid biosynthesis (v3.5) | 231 | 254998_at |
| 0.72 | AT1G66970 | glycerophosphodiester phosphodiesterase; | | 25 - defense response | 255852_at |

| | | | | | |
|---|---|---|---|---|---|
| | | glycerophosphodiester phosphodiesterase//kinase | | | |
| 0.72 | AT2G40880 | FL3-27; CYSTEINE PROTEINASE INHIBITOR | | 183 | 245096_at |
| 0.72 | AT4G14020 | | | 800 | 245385_at |
| 0.72 | AT4G24450 | kinase | | | 254153_at |
| 0.72 | AT1G12280 | | | 57 - phloem specific (vasculature tissues - specific) | 259535_at |
| 0.72 | AT5G52030 | | | 2 - photosynthe sis | 248391_at |
| 0.72 | AT2G41090 | CALCIUM BINDING; CALCIUM-BINDING | | 25 - defense response | 267076_at |
| 0.72 | AT4G25650 | oxidoreductase | | 2 - photosynthe sis | 254021_at |
| 0.72 | AT5G05200 | | | 2 - photosynthe sis | 250824_at |
| 0.71 | AT2G29670 | | | 537 | 266617_at |
| 0.71 | AT2G23200 | kinase | | 25 - defense response | 245074_at |
| 0.71 | AT1G28140 | CYTOCHROME OXIDASE | | 2 - photosynthe sis | 259594_at |
| 0.71 | AT1G76130 | AAM1; AAM2; AAM3; alpha amylase; ALPHA-AMYLASE; AtAAM1; AtAAM2; AtAAM3; SS3 | glycogen catabolism; starch degradation | 860 | 261754_at |
| 0.71 | AT1G69730 | kinase | | 25 - defense response | 260419_at |
| 0.71 | AT5G02600 | | | 57 - phloem specific (vasculature tissues - specific) | 251006_at |
| 0.71 | AT3G18500 | | | 2 - photosynthe sis | 257723_at |
| 0.71 | AT1G21065 | | | 2 - photosynthe sis | 261457_at |
| 0.71 | AT5G17450 | | | 497 | 246429_at |
| 0.71 | AT3G44880 | ACD1; ACCELERATED CELL DEATH 1; LLS1 | chlorophyll a degradation | 188 | 246335_at |
| 0.71 | AT2G32860 | hydrolase, hydrolyzing O-glycosyl compounds | | 731 | 267645_at |
| 0.71 | AT5G57040 | lactoylglutathione lyase | methylglyoxal degradation | 2 - photosynthe sis | 247931_at |

| | | | | | |
|---|---|---|---|---|---|
| 0.71 | AT1G68585 | | | 25 - defense response | 262286_at |
| 0.71 | AT4G27390 | | | 143 | 253893_at |
| 0.71 | AT5G46270 | | | 57 - phloem specific (vasculature tissues - specific) | 248890_at |
| 0.70 | AT5G23240 | | | 230 | 249850_at |
| 0.70 | AT1G67970 | AT-HSFA8; HSFA8 | | 463 | 259992_at |
| 0.70 | AT4G29670 | THIOREDOXIN; thiol-disulfide exchange intermediate | | 245 | 253693_at |
| 0.70 | AT4G11890 | | | 10 - protein modification , defense response | 254869_at |
| 0.70 | AT3G62550 | ER6 | | 350 | 251221_at |
| 0.70 | AT3G20300 | | | 1 - mixed (tricellular and mature pollen - specific) | 257672_at |
| 0.70 | AT2G29630 | | thiamin biosynthesis | 2 - photosynthe sis | 266673_at |
| 0.70 | AT2G46600 | | | 986 | 265460_at |
| 0.70 | AT2G24160 | catalytic | | 35 - kinases, signaling, disease resistance | 265993_at |
| 0.70 | AT1G56150 | | | | 262092_at |
| 0.70 | AT4G27990 | | | 914 | 253857_at |
| 0.70 | AT1G59620 | CW9 | | 25 - defense response | 262126_at |
| 0.70 | | | | 2 - photosynthe sis | 266599_at |
| 0.70 | AT4G23180 | CRK10; CYSTEINE-RICH RLK10; RECEPTOR-LIKE PROTEIN KINASE 4; RLK4 | | 578 | 254256_at |
| 0.70 | AT3G26580 | | | 143 | 257611_at |
| 0.70 | AT1G77930 | | | 15 - plastid stress and circadian rhythm | 262194_at |
| 0.70 | AT5G38250; AT5G38240 | | | 216 | 249552_s _at |

| | | | | | |
|---|---|---|---|---|---|
| 0.70 | AT2G24850 | TAT3; TAT; TYROSINE AMINOTRANSFERASE; TYROSINE AMINOTRANSFERASE 3; tyrosine transaminase | JA signalling; phenylalanine biosynthesis II; tyrosine degradation; tyrosine degradation 1 | 348 | 263539_at |
| 0.70 | AT2G25510 | | | 25 - defense response | 265611_at |
| 0.70 | AT3G60420 | | | 67 - Ca 2+ - triggered exocytosis (pathogen response?) | 251400_at |
| 0.70 | AT2G29320 | TROPINONE REDUCTASE; oxidoreductase | | 139 | 266291_at |
| 0.70 | AT1G06650 | OXIDOREDUCTASE IRON/ASCORBATE OXIDOREDUCTASE; oxidoreductase, acting on paired donors, with incorporation or reduction of molecular oxygen, 2-oxoglutarate as one donor, and incorporation of one atom each of oxygen into both donors | glucosinolate biosynthesis from homomethionine | 559 | 262638_at |
| 0.70 | AT5G03880 | | | 2 - photosynthesis | 250867_at |
| 0.70 | AT5G19140 | | | 231 | 249922_at |

**Table S4. Genes negatively correlated to the *SAQR* transcript profile.** Genes negatively correlated by Spearman's correlation (≤ -0.6). Pathway and regulon data are derived from AtGeneSearch (http://www.metnetdb.org/MetNet_atGeneSearch.htm) (Mentzen and Wurtele, 2008; Sucaet et al., 2012). mRNA transcriptome profiling dataset ("At956-2008", from 956 Affymetrix ATH1 chips) was used (Li et al., 2007; Mentzen and Wurtele, 2008; Li et al., 2009).

| Correlation | Locus ID | Gene | Pathways | Regulons | Probe Name |
|---|---|---|---|---|---|
| -0.74 | AT1G79530; AT1G16300 | | | 114 | 262939_s_at |
| -0.74 | AT1G14900 | | | 4 - mitosis | 262840_at |
| -0.74 | AT5G27450 | MEVALONATE KINASE; galactokinase; phosphomevalonate kinase | galactose degradation I; galactose metabolism; galactose, galactoside and glucose catabolism; isoprenoid (WYJ); lactose degradation 4; mevalonate pathway; mevalonate pathway (v4.0) | 166 | 246778_at |
| -0.72 | AT5G23530 | | | | 249794_at |
| -0.72 | AT2G26760 | CYCB1;4; CYC3; CYCLIN; CYCLIN 3; CYCLIN B1;4 | | 4 - mitosis | 267618_at |
| -0.71 | AT5G55480 | glycerophosphodiester phosphodiesterase | | 162 | 248085_at |
| -0.70 | AT3G48150 | APC8; CDC23 | | 3 - protein synthesis | 252349_at |
| -0.70 | AT4G05190 | ATK5 | | 4 - mitosis | 255265_at |
| -0.70 | AT3G25980 | | | 4 - mitosis | 258067_at |
| -0.70 | AT3G04810 | | | 4 - mitosis | 259083_at |
| -0.69 | AT4G25240 | SKS1; SKU5 SIMILAR 1 | | 4 - mitosis | 254109_at |
| -0.69 | AT1G02690 | | | 4 - mitosis | 260910_at |
| -0.69 | AT2G17370 | HMG2; Hydroxymethylglutaryl-CoA reductase (NADPH); AT2G17370; hydroxymethylglutaryl-CoA reductase | isoprenoid (WYJ); mevalonate degradation | 1 - mixed (tricellular and mature pollen - specific) | 264856_at |
| -0.69 | AT5G43060 | CYSTEINE PROTEASE; G91-MONOMER | | 1 - mixed (tricellular and mature | 249187_at |

| | | | | | | |
|---|---|---|---|---|---|---|
| | | | | pollen - specific) | | |
| -0.69 | AT1G74030 | ENOLASE; phosphopyruvate hydratase | aerobic glycerol catabolism; gluconeogenesis; glycolysis I; glycolysis II; glycolysis I (plant cytosol); glycolysis II (plant plastids) (v4.0); glycolysis II (plant plastids); Glycolysis-cytosol (WYJ); serine-isocitrate lyase pathway; sucrose degradation to ethanol and lactate (anaerobic) | 114 | 260392_at |
| -0.69 | AT1G76540 | CDKB2;1; Cyclin-dependent kinase B2;1 | | 4 - mitosis | 259978_at |
| -0.69 | AT1G49580 | kinase | | 162 | 261605_at |
| -0.68 | AT5G41060 | | | 162 | 249290_at |
| -0.68 | AT5G49460 | ACLB-2; ACLB; ATP CITRATE LYASE; ATP-CITRATE-LYASE; citrate (pro-3S)-lyase | acetyl-CoA biosynthesis (from citrate); Acetyl-CoA Biotin network; superpathway of acetyl-CoA biosynthesis | 167 | 248608_at |
| -0.68 | AT3G16490 | | | 4 - mitosis | 257229_at |
| -0.68 | AT2G42570 | | | 484 | 263496_at |
| -0.68 | AT1G50490 | UBC20 | | 4 - mitosis | 261859_at |
| -0.68 | AT5G37010 | | | 4 - mitosis | 249644_at |
| -0.67 | AT5G03670 | | | | 250907_at |
| -0.67 | AT1G71830 | SERK1; ATSERK1; SOMATIC EMBRYOGENESIS RECEPTOR-LIKE KINASE 1 | | 4 - mitosis | 261521_at |

| | | | | | |
|---|---|---|---|---|---|
| -0.67 | AT5G54670 | ATK3; ARABIDOPSIS THALIANA KINESIN 3; KATC; KINESIN; KINESIN-LIKE PROTEIN C; ATPase | | 4 - mitosis | 248150_at |
| -0.67 | AT4G37590 | | | | 253062_at |
| -0.66 | AT3G01330 | DEL3; DP-E2F-LIKE PROTEIN 3 | AGRIS regulatory network - full | 1 - mixed (tricellular and mature pollen - specific) | 257524_at |
| -0.66 | AT2G25880 | | | 1 - mixed (tricellular and mature pollen - specific) | 266655_at |
| -0.66 | AT2G35120 | GLYCINE DECARBOXYLASE COMPLEX H-PROTEIN; glycine decarboxylase H protein; glycine decarboxylase H protein~MONOMER-907; glycine dehydrogenase (decarboxylating) | folate transformations; glycine degradation; photorespiration; formylTHF biosynthesis | 114 | 266517_at |
| -0.66 | AT5G23400 | | | 162 | 249832_at |
| -0.66 | AT5G23430 | | | 550 | 249833_at |
| -0.66 | AT1G77720 | | | 4 - mitosis | 259679_at |
| -0.66 | AT1G76550 | PYROPHOSPHATE-DEPENDENT PHOSPHOFRUCTOKINASE SUBUNIT ALPHA; 6-phosphofructokinase | glycolysis I (plant cytosol) | 167 | 259969_at |
| -0.65 | AT5G17160 | | | 4 - mitosis | 246415_at |
| -0.65 | AT2G38620; AT3G54180 | | | 4 - mitosis | 266401_s_at |
| -0.65 | AT5G62410 | SMC2; ATSMC4; SMC4; STRUCTURAL MAINTENANCE OF CHROMOSOMES 2; TITAN3; TTN3 | AGRIS regulatory network - full | 4 - mitosis | 247482_at |
| -0.65 | AT2G34710 | PHB; ATHB-14; ATHB14; PHABULOSA; PHB-1D | | 16 - information | 267316_at |
| -0.65 | AT1G20590; AT1G20610 | | | 4 - mitosis | 259563_s_at |
| -0.65 | AT3G14190 | | | 4 - mitosis | 257005_at |
| -0.65 | AT5G57970 | DNA-3-METHYLADENINE GLYCOSYLASE | | 317 | 247892_at |
| -0.65 | AT4G22130 | | | 317 | 254341_at |
| -0.65 | AT5G44560 | | | 4 - mitosis | 249060_at |
| -0.65 | AT1G04030 | | | | 265096_at |

| | | | | | |
|---|---|---|---|---|---|
| -0.65 | AT3G16620 | ATTOC120 | | 550 | 258429_at |
| -0.65 | AT2G45490 | | | 1 - mixed (tricellular and mature pollen - specific) | 267529_at |
| -0.65 | AT1G32090 | | | 449 | 245789_at |
| -0.65 | AT3G53240 | | | | 251976_at |
| -0.65 | AT3G05020 | ACP1; ACP; ACYL CARRIER PROTEIN | | 167 | 259095_at |
| -0.65 | AT1G76310 | CYCB2;4; CYCLIN B2;4 | | 4 - mitosis | 261780_at |
| -0.64 | AT1G08560 | SYP111; ATSYP111; KN; KNOLLE; SYNTAXIN | | 4 - mitosis | 264802_at |
| -0.64 | AT5G48310 | | | 4 - mitosis | 248691_at |
| -0.64 | AT1G12000 | PFP-B; pyrophosphate-dependent fructose bisphosphatase B subunit; pyrophosphate-dependent PFK; pyrophosphate-dependent phosphofructokinase; PYROPHOSPHATE-FRUCTOSE-6-PHOSPHATE 1-PHOSPHOTRANSFERASE; 6-phosphofructokinase | glycolysis I; glycolysis I (plant cytosol) | 650 | 264386_at |
| -0.64 | AT5G11020 | | | 162 | 245898_at |
| -0.64 | AT5G01660 | | | 4 - mitosis | 251099_at |
| -0.64 | AT2G27510 | FERREDOXIN | | 114 | 265649_at |
| -0.64 | AT5G62550 | | | 47 - nuclear, replication, chromosome organization, cell cycle | 247425_at |
| -0.64 | AT3G50620 | NODH | | | 252164_at |
| -0.64 | AT1G11670 | | | 5 - membrane transporters –metal, toxins removal (root - preferential) | 262813_at |
| -0.63 | AT5G04770 | CAT6; CATIONIC AMINO ACID TRANSPORTER 6 | | 9 - response to environmetal stimula | 250860_at |
| -0.63 | AT1G44110 | CYCA1;1; Cyclin A1;1; CYCLIN A2-TYPE | | 4 - mitosis | 245739_at |

| | | | | | |
|---|---|---|---|---|---|
| -0.63 | AT4G32830 | | | 1 - mixed (tricellular and mature pollen - specific) | 253403_at |
| -0.63 | AT2G47630 | PHOSPHOLIPASE; catalytic | | | 266485_at |
| -0.63 | AT5G48230 | Acetoacetyl-CoA thiolase; ACETYL-COA C-ACETYLTRANSFERASE; EMB1276; EMBRYO DEFECTIVE 1276 | isoprenoid (WYJ); lysine degradation II; lysine degradation III; mevalonate pathway; mevalonate pathway (v4.0); tryptophan degradation; tryptophan degradation III | 166 | 248690_at |
| -0.63 | AT2G30050 | | | | 267277_at |
| -0.63 | AT1G32760 | | | | 261702_at |
| -0.63 | AT4G03100 | | | 4 - mitosis | 255410_at |
| -0.63 | AT3G12870 | OXIDOREDUCTASES | | 4 - mitosis | 257134_at |
| -0.63 | AT1G71010 | 1-phosphatidylinositol-4-phosphate 5-kinase | | 829 | 262308_at |
| -0.63 | AT2G01210 | | | 4 - mitosis | 265789_at |
| -0.63 | AT5G10540 | OLIGOPEPTIDASE A | | 16 - information | 250441_at |
| -0.63 | AT4G38210 | ATEXPA20; ARABIDOPSIS THALIANA EXPANSIN A20; ATEXP20; ATHEXP ALPHA 1.23; EXP20; GHEX1 | | | 253008_at |
| -0.63 | AT1G66040; AT1G66050; AT5G39550 | | | 64 - information, nuclear | 249457_s_at |
| -0.63 | AT1G78770 | | | 4 - mitosis | 264293_at |
| -0.63 | AT5G06970 | | | | 250656_at |
| -0.63 | AT5G15510 | | AGRIS regulatory network - full | 4 - mitosis | 246557_at |
| -0.63 | AT4G15830 | | | 4 - mitosis | 245343_at |
| -0.63 | AT4G35730 | | | 4 - mitosis | 253156_at |
| -0.63 | AT4G14150 | PAKRP1; ARPC4; PHRAGMOPLAST-ASSOCIATED KINESIN-RELATED PROTEIN 1 | | 4 - mitosis | 245259_at |
| -0.62 | AT3G51280 | MS5 | | 4 - mitosis | 252148_at |
| -0.62 | AT3G23890 | TOPII; TOPOISOMERASE; TOPOISOMERASE II | | 4 - mitosis | 256864_at |

| | | | | |
|---|---|---|---|---|
| -0.62 | AT5G20540 | | 16 - information | 246085_at |
| -0.62 | AT1G52250 | DYNEIN | 1 - mixed (tricellular and mature pollen - specific) | 257504_at |
| -0.62 | AT2G07170 | | 4 - mitosis | 266427_at |
| -0.62 | AT1G76760 | THIOREDOXIN; isomerase//thiol-disulfide exchange intermediate | 343 | 259868_at |
| -0.62 | AT1G53140 | DYNAMIN | 4 - mitosis | 261364_at |
| -0.62 | AT1G50240 | | 4 - mitosis | 262467_at |
| -0.62 | AT5G01620 | | 166 | 251108_at |
| -0.62 | AT2G21060 | ATGRP2B; GLYCINE-RICH PROTEIN 2B | | 264026_at |
| -0.62 | AT3G50960 | | 13 - upregulate d in 'response to CO2 levels' experiment | 252127_at |
| -0.62 | AT5G61980 | | 1 - mixed (tricellular and mature pollen - specific) | 247503_at |
| -0.61 | AT1G69400 | | | 260349_at |
| -0.61 | AT5G33300 | CHROMOKINESIN | 91 | 246683_at |
| -0.61 | AT2G42110 | | 4 - mitosis | 267636_at |
| -0.61 | AT1G72730 | | 8 - information (uninucleat e microspore and bicellular pollen- specific) | 259891_at |
| -0.61 | AT5G16250 | | 4 - mitosis | 246505_at |
| -0.61 | AT1G63640 | | 1 - mixed (tricellular and mature pollen - specific) | 261557_at |
| -0.61 | AT5G66230 | | | 247134_at |
| -0.61 | AT2G27970 | CDK-subunit 2; CKS2; CYCLIN-DEPENDENT KINASE REGULATORY | 64 - information, nuclear | 264061_at |

| | | | | | |
|---|---|---|---|---|---|
| -0.61 | AT3G03320 | | | 6 - embryo maturation (fruit and seed preferential ) | 259053_at |
| -0.61 | AT4G22120 | | | | 254340_at |
| -0.61 | AT2G22620 | lyase | | 12 - mixed (fruit - preferential ) | 265350_at |
| -0.61 | AT4G39860 | | | 4 - mitosis | 252821_at |
| -0.61 | AT5G65020 | ANNAT2; ANNEXIN | | 5 - membrane transporter s –metal, toxins removal (root - preferential ) | 247210_at |
| -0.61 | AT4G33270; AT4G33260 | | | 4 - mitosis | 253340_s _at |
| -0.61 | AT3G03130 | | | 4 - mitosis | 258867_at |
| -0.61 | AT4G05520 | | | 4 - mitosis | 255236_at |
| -0.61 | AT2G36200 | KINESIN-RELATED CYTOKINESIS | | 4 - mitosis | 263960_at |
| -0.61 | AT5G03150 | | | 9 - response to environmet al stimula | 250982_at |
| -0.61 | AT2G33560 | | | | 255790_at |
| -0.60 | AT4G37490 | CYC1; CYCB1; CYCB1;1; CYCLIN 1; Cyclin B1;1 | AGRIS regulatory network - full | 149 | 253051_at |
| -0.60 | AT5G60200 | | | 882 | 247625_at |
| -0.60 | AT5G38110 | | AGRIS regulatory network - full | 47 - nuclear, replication, chromosom e organizatio n, cell cycle | 249544_at |
| -0.60 | AT3G11760 | | | 1 - mixed (tricellular and mature pollen - specific) | 258783_at |
| -0.60 | AT2G43040 | NPG1; NO POLLEN GERMINATION 1 | | 1 - mixed (tricellular and mature pollen - specific) | 265243_at |

| | | | | | |
|---|---|---|---|---|---|
| -0.60 | AT3G22880 | ATDMC1; DMC1; RECA-LIKE GENE | | | 256832_at |
| -0.60 | AT1G20930 | CDKB2;2; Cyclin-dependent kinase B2;2; kinase | | 4 - mitosis | 262802_at |
| -0.60 | AT5G46700 | TRN2 | | 16 - information | 248861_at |
| -0.60 | AT5G65640 | | | | 247151_at |
| -0.60 | AT4G14290 | | | | 245604_at |
| -0.60 | AT5G51380 | | | | 248456_at |
| -0.60 | AT2G36290 | | | 5 - membrane transporters –metal, toxins removal (root - preferential) | 263900_at |
| -0.60 | AT5G14000 | ANAC084 | | 7 - developmental regulation (leaf apex-preferential) | 250208_at |
| -0.60 | AT3G25900 | HOMOCYSTEINE S-METHYLTRANSFERASE | methionine biosynthesis; S-methylmethionine cycle; superpathway of lysine, threonine, and methionine biosynthesis | 1 - mixed (tricellular and mature pollen - specific) | 258075_at |
| -0.60 | AT4G24610 | | | 4 - mitosis | 254140_at |
| -0.60 | AT5G19340 | | | 665 | 246063_at |
| -0.60 | AT3G56640 | EXOCYST COMPLEX; hydrolase, hydrolyzing O-glycosyl compounds | | 124 | 251700_at |
| -0.60 | AT4G17610 | | | 4 - mitosis | 245404_at |
| -0.60 | AT3G19100 | | | | 257024_at |
| -0.60 | AT1G68120 | | | 1 - mixed (tricellular and mature pollen - specific) | 259998_at |
| -0.60 | AT2G17560 | HMGB4; HIGH MOBILITY GROUP B 4; HMG GAMMA; NFD04; NFD4 | | 4 - mitosis | 263074_at |
| -0.60 | AT5G13520 | LEUKOTRIENE-A4 HYDROLASE | gamma-glutamyl cycle | 4 - mitosis | 245849_at |

| | | | | |
|---|---|---|---|---|
| -0.60 | At4g30850 | | 1 - mixed (tricellular and mature pollen - specific) | 253590_at |
| -0.60 | AT1G03780 | | 4 - mitosis | 265085_at |

**Table S5. Positive correlation of senescence-associated genes (SAGs) with *SAQR*.** One hundred and thirty three genes are positively co-expressed with *SAQR* (correlation coefficient ≥0.7 with *SAQR*) across multiple conditions. Forty percent of these (53 genes) are in the subset of SAGs that are up-regulated under natural senescence but not induced senescence. #SAG data from (Van der Graaff et al., 2006). ##mRNA transcriptome profiling dataset ("At956-2008", from 956 Affymetrix ATH1 chips) was used (Li et al., 2007; Mentzen and Wurtele, 2008; Li et al., 2009).

| **Condition#** | **Total number of SAGs up-regulated** | **Number of SAGs positively correlated with *SAQR*** | **% in genes positively correlated with *SAQR*##** |
|---|---|---|---|
| Natural senescence (NS) | 1045 | 53 | 40 |
| Natural senescence (NS); Induced attached (DIS); Induced detached (DET) | 504 | 3 | 2 |
| Natural senescence (NS); Induced attached (DIS) | 120 | 2 | 1 |
| Natural senescence (NS); Induced detached (DET) | 286 | 0 | -- |
| Induced detached (DET) | 160 | 0 | -- |
| Induced attached (DIS) | 116 | 0 | -- |
| Induced attached (DIS); Induced detached (DET) | 81 | 0 | -- |

| **Condition#** | **Total number of SAGs down-regulated** | **Number of SAGs positively correlated with *SAQR*** | **% in genes positively correlated with *SAQR*##** |
|---|---|---|---|
| Natural senescence (NS); Induced attached (DIS); Induced detached (DET) | 726 | 4 | 3 |
| Natural senescence (NS); Induced detached (DET) | 191 | 3 | 2 |
| Natural senescence (NS); Induced attached (DIS) | 137 | 0 | - |
| Natural senescence (NS) | 501 | 2 | 1 |
| Induced detached (DET) | 139 | 6 | 4 |
| Induced attached (DIS); Induced detached (DET) | 56 | 6 | 4 |
| Induced attached (DIS) | 96 | 2 | 1 |

**Table S6. Negative correlation of senescence-associated genes (SAGs) with *SAQR*.** One hundred and thirty-four genes are negatively correlated with *SAQR* (correlation coefficient ≤-0.6 with *SAQR*) across multiple conditions. Only eight of these genes are SAGs. #SAG data from (Van der Graaff et al., 2006). ##mRNA transcriptome profiling dataset ("At956-2008", from 956 Affymetrix ATH1 chips) was used (Li et al., 2007; Mentzen and Wurtele, 2008; Li et al., 2009).

| **Condition#** | **Total number of SAGs up-regulated** | **Number of SAGs negatively correlated with *SAQR*** | **% in genes negatively correlated with *SAQR*##** |
|---|---|---|---|
| Natural senescence (NS) | 1045 | 5 | 4 |
| Natural senescence (NS); Induced attached (DIS); Induced detached (DET) | 504 | 1 | <1 |
| Natural senescence (NS); Induced attached (DIS) | 120 | 0 | - |
| Natural senescence (NS); Induced detached (DET) | 286 | 0 | - |
| Induced detached (DET) | 160 | 2 | 1 |
| Induced attached (DIS) | 116 | 0 | - |
| Induced attached (DIS); Induced detached (DET) | 81 | 0 | - |

| **Condition#** | **Total # SAGs down-regulated in condition** | **SAGs negatively correlated with *SAQR*** | **% in genes negatively correlated with *SAQR*##** |
|---|---|---|---|
| Natural senescence (NS); Induced attached (DIS); Induced detached (DET) | 726 | 1 | <1 |
| Natural senescence (NS); Induced detached (DET) | 191 | 0 | - |
| Natural senescence (NS); Induced attached (DIS) | 137 | 0 | - |
| Natural senescence (NS) | 501 | 2 | 1 |
| Induced detached (DET) | 139 | 0 | - |
| Induced attached (DIS); Induced detached (DET) | 56 | 0 | - |
| Induced attached (DIS) | 96 | 0 | - |

**Table S7. Genes with altered expression profiles in *saqr* mutants.** Data represents *P* values, Q values, and fold change comparing the SALK_052233C KO (*saqr*) and wild type lines. Categorical information and annotations were gleaned from MetNetDB. Genes with *P* value ≤ 0.01 are listed here.

| Locus ID | TAIR Annotation | Gene Name | Category | *P* value | Q value | Fold Change (*saqr* vs WT) |
|---|---|---|---|---|---|---|
| AT1G64360 | unknown protein | SAQR | N/A | 0.0000000001 | 0.000002 | 0.0000000006 |
| **AT1G22900** | Disease resistance-responsive (dirigent-like protein) family protein | Disease resistance-responsive (dirigent-like protein) family protein; | Defense | 0.00001 | 0.13 | 7.165 |
| AT3G22840 | EARLY LIGHT-INDUCABLE PROTEIN (ELIP1) | Chlorophyll A-B binding family protein; | Light response | 0.00002 | 0.13 | 0.282 |
| AT2G44790 | uclacyanin 2 (UCC2) | toxins removal (root - preferential); | Transport | 0.0001 | 0.55 | 5.168 |
| AT4G22510 | unknown protein | | N/A | 0.0002 | 0.55 | 4.181 |
| AT3G50410 | OBF binding protein 1 (OBP1) | OBF binding protein 1; | Transcription factor | 0.0002 | 0.55 | 4.739 |
| AT1G30250 | unknown protein | | N/A | 0.0002 | 0.55 | 0.360 |
| AT2G01850 | endoxyloglucan transferase A3 (EXGT-A3) | endoxyloglucan transferase A3; | Sugar synthesis | 0.0002 | 0.55 | 2.244 |
| AT1G72430 | SAUR-like auxin-responsive protein family | SAUR-like auxin-responsive protein family; | Hormone (Auxin) | 0.0003 | 0.70 | 2.343 |
| AT5G67420 | LOB domain-containing protein 37 (LBD37) | LOB domain-containing protein 37; | N/A | 0.0004 | 0.70 | 0.602 |
| AT3G16660 | Pollen Ole e 1 allergen and extensin family protein | Pollen Ole e 1 allergen and extensin family protein; | N/A | 0.0004 | 0.70 | 3.084 |
| AT1G20990 | Cysteine/Histidine-rich C1 domain family protein | Cysteine/Histidine-rich C1 domain family protein; | Oxidation-reduction | 0.0004 | 0.70 | 0.074 |
| AT3G58760 | Integrin-linked protein kinase family | Integrin-linked protein kinase family; | Signaling transduction | 0.0005 | 0.73 | 0.608 |
| AT4G22517 | Bifunctional inhibitor/lipid-transfer protein/seed storage 2S albumin superfamily | Bifunctional inhibitor/lipid-transfer protein/seed storage 2S albumin superfamily protein; | Transport | 0.0006 | 0.73 | 4.818 |
| AT3G11240 | arginine-tRNA protein transferase 2 (ATE2) | arginine-tRNA protein transferase 2; | Protein modification | 0.0006 | 0.73 | 0.358 |
| AT2G47880 | Glutaredoxin family protein | Glutaredoxin family protein; | Oxidation-reduction | 0.0007 | 0.79 | 2.555 |
| AT1G23030 | ARM repeat superfamily protein | ARM repeat superfamily protein; | Protein modification | 0.0007 | 0.79 | 1.836 |

| | | | | | | |
|---|---|---|---|---|---|---|
| AT1G07430 | highly ABA-induced PP2C gene 2 (HAI2) | highly ABA-induced PP2C gene 2; | Hormone (ABA) | 0.0008 | 0.82 | 0.222 |
| AT3G58120 | BZIP61 | Basic-leucine zipper (bZIP) transcription factor family protein; | Transcription factor | 0.0009 | 0.82 | 2.153 |
| AT5G56780 | effector of transcription2 (ET2) | effector of transcription2; | Transcription factor | 0.0009 | 0.82 | 3.907 |
| AT5G22430 | Pollen Ole e 1 allergen and extensin family protein | Pollen Ole e 1 allergen and extensin family protein; | N/A | 0.0009 | 0.82 | 0.000 |
| AT1G61370 | S-locus lectin protein kinase family protein | S-locus lectin protein kinase family protein; | Protein modification | 0.0010 | 0.82 | 0.339 |
| AT2G02770 | 4'-phosphopantetheinyl transferase superfamily | 4'-phosphopantetheinyl transferase superfamily; | N/A | 0.0010 | 0.82 | 2.178 |
| AT3G62720 | xylosyltransferase 1 (XT1) | xylosyltransferase 1; | Sugar synthesis | 0.0012 | 0.94 | 1.879 |
| AT5G62470 | myb domain protein 96 (MYB96) | myb domain protein 96; | Transcription factor | 0.0013 | 0.98 | 0.538 |
| AT2G28120 | Major facilitator superfamily protein | Major facilitator superfamily protein; | Transport | 0.0014 | 1.00 | 5.963 |
| AT5G48290 | Heavy metal transport/detoxification superfamily protein | toxins removal (root - preferential); | Transport | 0.0015 | 1.00 | 0.097 |
| AT1G24095 | Putative thiol-disulphide oxidoreductase DCC | Putative thiol-disulphide oxidoreductase DCC; | Oxidation-reduction | 0.0016 | 1.00 | 10.797 |
| AT5G06530 | ABC-2 type transporter family protein | ABC-2 type transporter family protein; | Oxidation-reduction | 0.0016 | 1.00 | 0.634 |
| AT1G10970 | zinc transporter 4 precursor (ZIP4) | zinc transporter 4 precursor; | Transport | 0.0016 | 1.00 | 4.339 |
| AT5G65650 | Protein of unknown function (DUF1195) | Protein of unknown function (DUF1195); | N/A | 0.0016 | 1.00 | 1.777 |
| AT3G47640 | POPEYE (PYE) | basic helix-loop-helix (bHLH) DNA-binding superfamily protein; | Transcription factor | 0.0018 | 1.00 | 0.492 |
| AT5G14120 | Major facilitator superfamily protein | Major facilitator superfamily protein; | Transport | 0.0018 | 1.00 | 1.734 |
| AT5G54160 | O-methyltransferase 1 (OMT1) | O-methyltransferase 1; | Secondary metabolite synthesis | 0.0019 | 1.00 | 1.461 |
| AT4G12490 | Bifunctional inhibitor/lipid-transfer protein/seed storage 2S albumin superfamily | Bifunctional inhibitor/lipid-transfer protein/seed storage 2S albumin superfamily protein; | Transport | 0.0022 | 1.00 | 3.943 |
| AT1G05065 | CLAVATA3/ESR-RELATED 20 (CLE20) | CLAVATA3/ESR-RELATED 20; | Signaling transduction | 0.0022 | 1.00 | 0.082 |
| AT5G19300 | CONTAINS InterPro DOMAIN/s: | | N/A | 0.0022 | 1.00 | 2.069 |

| | | | | | | |
|---|---|---|---|---|---|---|
| | Nucleic acid-binding, OB-fold-like (InterPro:IPR016027), | | | | | |
| AT5G37260 | REVEILLE 2 (RVE2) | Homeodomain-like superfamily protein; | Transcription factor | 0.0024 | 1.00 | 0.656 |
| AT3G11220 | ELONGATA 1 (ELO1) | Paxneb protein-related; | Abiotic stress response | 0.0024 | 1.00 | 1.775 |
| AT3G54810 | BLUE MICROPYLAR END 3 (BME3) | Plant-specific GATA-type zinc finger transcription factor family protein; | Transcription factor | 0.0024 | 1.00 | 1.539 |
| AT1G45110 | Tetrapyrrole (Corrin/Porphyrin) Methylases | Tetrapyrrole (Corrin/Porphyrin) Methylases; | Nucleic acid modification | 0.0026 | 1.00 | 3.463 |
| AT2G20870 | cell wall protein precursor, putative | cell wall protein precursor; | N/A | 0.0027 | 1.00 | 0.323 |
| AT4G19810 | Glycosyl hydrolase family protein with chitinase insertion domain | Glycosyl hydrolase family protein with chitinase insertion domain; | Abiotic stress response | 0.0028 | 1.00 | 3.160 |
| AT3G24520 | heat shock transcription factor C1 (HSFC1) | heat shock transcription factor C1; | Transcription factor | 0.0029 | 1.00 | 0.417 |
| AT5G62210 | Embryo-specific protein 3, (ATS3) | Embryo-specific protein 3; | N/A | 0.0030 | 1.00 | 0.577 |
| AT5G24380 | YELLOW STRIPE like 2 (YSL2) | YELLOW STRIPE like 2; | Transport | 0.0030 | 1.00 | 0.464 |
| AT5G19110 | Eukaryotic aspartyl protease family protein | Eukaryotic aspartyl protease family protein; | Protein modification | 0.0031 | 1.00 | 4.619 |
| AT3G51450 | Calcium-dependent phosphotriesterase superfamily protein | Calcium-dependent phosphotriesterase superfamily protein; | Hormone (Jasmonate) | 0.0032 | 1.00 | 1.939 |
| AT1G70300 | K+ uptake permease 6 (KUP6) | K+ uptake permease 6; | Transport | 0.0032 | 1.00 | 2.987 |
| AT5G37300 | WSD1 | O-acyltransferase (WSD1-like) family protein; | Lipid synthesis | 0.0032 | 1.00 | 0.390 |
| AT1G15125 | S-adenosyl-L-methionine-dependent methyltransferases superfamily protein | S-adenosyl-L-methionine-dependent methyltransferases superfamily protein; | Protein modification | 0.0033 | 1.00 | 3.184 |
| AT2G38250 | Homeodomain-like superfamily protein | Homeodomain-like superfamily protein; | Transcription factor | 0.0033 | 1.00 | 4.413 |
| AT2G30020 | Protein phosphatase 2C family protein | Protein phosphatase 2C family protein; | Abiotic stress response | 0.0033 | 1.00 | 0.550 |
| AT5G65760 | Serine carboxypeptidase S28 family protein | Serine carboxypeptidase S28 family protein; | Catabolism | 0.0034 | 1.00 | 1.728 |
| AT3G16770 | ethylene-responsive | ethylene-responsive element binding protein; | Transcription factor | 0.0034 | 1.00 | 0.545 |

| | | | | | | |
|---|---|---|---|---|---|---|
| | element binding protein (EBP) | | | | | |
| AT1G76160 | SKU5 similar 5 (sks5) | SKU5 similar 5; | Secondary metabolite synthesis | 0.0035 | 1.00 | 1.411 |
| AT1G64500 | Glutaredoxin family protein | Glutaredoxin family protein; | Oxidation-reduction | 0.0035 | 1.00 | 0.643 |
| AT3G46900 | copper transporter 2 (COPT2) | copper transporter 2; | Transport | 0.0037 | 1.00 | 0.444 |
| AT4G39780 | Integrase-type DNA-binding superfamily protein | Integrase-type DNA-binding superfamily protein; | Transcriptio n factor | 0.0038 | 1.00 | 0.534 |
| AT2G32160 | S-adenosyl-L-methionine-dependent methyltransferas es superfamily protein | S-adenosyl-L-methionine-dependent methyltransferases superfamily protein; | Protein modificatio n | 0.0038 | 1.00 | 0.625 |
| AT1G21120 | O-methyltransferas e family protein | O-methyltransferase family protein; | Secondary metabolite synthesis | 0.0038 | 1.00 | 0.508 |
| AT1G18080 | ATARCA | Transducin/WD40 repeat-like superfamily protein; | Catabolism | 0.0040 | 1.00 | 1.492 |
| AT2G43590 | Chitinase family protein | Chitinase family protein; | Defense | 0.0042 | 1.00 | 3.028 |
| AT3G58520 | Ubiquitin carboxyl-terminal hydrolase family protein | Ubiquitin carboxyl-terminal hydrolase family protein; | Protein modificatio n | 0.0042 | 1.00 | 1.990 |
| AT2G43580 | Chitinase family protein | Chitinase family protein; | Defense | 0.0045 | 1.00 | 14.874 |
| AT3G13020 | hAT transposon superfamily protein | hAT transposon superfamily protein; | Protein modificatio n | 0.0045 | 1.00 | 64911692 |
| AT2G41230 | unknown protein | | N/A | 0.0046 | 1.00 | 4.206 |
| AT5G62280 | Protein of unknown function (DUF1442) | Protein of unknown function (DUF1442); | N/A | 0.0046 | 1.00 | 0.000 |
| AT2G16660 | Major facilitator superfamily protein | Major facilitator superfamily protein; | Transport | 0.0046 | 1.00 | 1.857 |
| AT1G23480 | cellulose synthase-like A3 (CSLA03) | cellulose synthase-like A3; | Sugar synthesis | 0.0046 | 1.00 | 1.894 |
| AT3G62960 | Thioredoxin superfamily protein | Thioredoxin superfamily protein; | Oxidation-reduction | 0.0048 | 1.00 | 282449502 |
| AT5G23020 | 2-isopropylmalate synthase 2 (IMS2) | toxins removal (root - preferential); | Secondary metabolite synthesis | 0.0048 | 1.00 | 1.861 |
| AT3G46880 | unknown protein | | N/A | 0.0049 | 1.00 | 6.156 |
| AT5G48655 | RING/U-box superfamily protein | RING/U-box superfamily protein; | Transport | 0.0050 | 1.00 | 0.573 |
| AT4G15800 | ralf-like 33 (RALFL33) | ralf-like 33; | Signaling transductio n | 0.0050 | 1.00 | 1.839 |
| AT4G35420 | dihydroflavonol 4-reductase-like1 (DRL1) | secondary wall (flower - specific; | Secondary metabolite synthesis | 0.0050 | 1.00 | 3.491 |
| AT3G15115 | unknown protein | | N/A | 0.0050 | 1.00 | 0.355 |

| | | | | | | |
|---|---|---|---|---|---|---|
| AT2G38310 | PYR1-like 4 (PYL4) | PYR1-like 4; | N/A | 0.0051 | 1.00 | 1.699 |
| AT2G36650 | unknown protein | | N/A | 0.0051 | 1.00 | 0.000 |
| AT1G75200 | flavodoxin family protein / radical SAM domain-containing protein | translation; | Oxidation-reduction | 0.0052 | 1.00 | 1.510 |
| AT1G60190 | ARM repeat superfamily protein | ARM repeat superfamily protein; | Protein modificatio n | 0.0052 | 1.00 | 0.298 |
| AT3G05730 | LOCATED IN: endomembrane system | | N/A | 0.0052 | 1.00 | 2.610 |
| AT4G08770 | Peroxidase superfamily protein | toxins removal (root - preferential); | Catabolism | 0.0052 | 1.00 | 7.453 |
| AT4G39950 | cytochrome P450, family 79, subfamily B, polypeptide 2 (CYP79B2) | cytochrome P450; | Catabolism | 0.0053 | 1.00 | 1.622 |
| AT2G17470 | Aluminium activated malate transporter family protein | Aluminium activated malate transporter family protein; | Transport | 0.0054 | 1.00 | 3.174 |
| AT1G64170 | cation/H+ exchanger 16 (CHX16) | defense response; | Transport | 0.0056 | 1.00 | 2.500 |
| AT3G27250 | unknown protein | | N/A | 0.0057 | 1.00 | 0.401 |
| AT1G02640 | beta-xylosidase 2 (BXL2) | beta-xylosidase 2; | Catabolism | 0.0058 | 1.00 | 2.260 |
| AT1G70090 | glucosyl transferase family 8 (LGT8) | glucosyl transferase family 8; | Sugar synthesis | 0.0059 | 1.00 | 1.657 |
| AT1G02870 | FUNCTIONS IN: molecular_functi on unknown | | N/A | 0.0059 | 1.00 | 1.727 |
| AT3G24750 | unknown protein | | N/A | 0.0061 | 1.00 | 0.117 |
| AT3G15210 | ethylene responsive element binding factor 4 (ERF4) | ethylene responsive element binding factor 4; | Transcriptio n factor | 0.0061 | 1.00 | 0.537 |
| AT1G76090 | sterol methyltransferas e 3 (SMT3) | sterol methyltransferase 3; | Secondary metabolite synthesis | 0.0061 | 1.00 | 1.783 |
| AT3G19680 | Protein of unknown function (DUF1005) | Protein of unknown function (DUF1005); | N/A | 0.0061 | 1.00 | 2.269 |
| AT1G06440 | Ubiquitin carboxyl-terminal hydrolase family protein | Ubiquitin carboxyl-terminal hydrolase family protein; | Protein modificatio n | 0.0063 | 1.00 | 3.467 |
| AT4G37260 | myb domain protein 73 (MYB73) | myb domain protein 73; | Transcriptio n factor | 0.0064 | 1.00 | 1.728 |
| AT5G63160 | BTB and TAZ domain protein 1 (BT1) | BTB and TAZ domain protein 1; | Transcriptio n factor | 0.0064 | 1.00 | 0.596 |
| AT4G16260 | Glycosyl hydrolase superfamily protein | toxins removal (root - preferential); | Defense | 0.0065 | 1.00 | 3.470 |

| | | | | | | |
|---|---|---|---|---|---|---|
| AT1G08030 | tyrosylprotein sulfotransferase (TPST) | tyrosylprotein sulfotransferase; | Protein modification | 0.0067 | 1.00 | 1.754 |
| AT1G64380 | Integrase-type DNA-binding superfamily protein | Integrase-type DNA-binding superfamily protein; | Transcription factor | 0.0067 | 1.00 | 0.379 |
| AT3G27280 | prohibitin 4 (PHB4) | prohibitin 4; | Nucleic acid modification | 0.0067 | 1.00 | 1.827 |
| AT4G37410 | cytochrome P450, family 81, subfamily F, polypeptide 4 (CYP81F4) | cytochrome P450; | Catabolism | 0.0068 | 1.00 | 3.051 |
| AT5G43150 | unknown protein | | N/A | 0.0070 | 1.00 | 0.635 |
| AT1G22530 | PATELLIN 2 (PATL2) | PATELLIN 2; | Transport | 0.0073 | 1.00 | 1.583 |
| AT5G05600 | 2-oxoglutarate (2OG) and Fe(II)-dependent oxygenase superfamily protein | 2-oxoglutarate (2OG) and Fe(II)-dependent oxygenase superfamily protein; | Hormone (Jasmonate) | 0.0074 | 1.00 | 2.879 |
| AT1G01120 | 3-ketoacyl-CoA synthase 1 (KCS1) | 3-ketoacyl-CoA synthase 1; | Lipid synthesis | 0.0074 | 1.00 | 1.508 |
| AT4G22513 | Bifunctional inhibitor/lipid-transfer protein/seed storage 2S albumin superfamily | Bifunctional inhibitor/lipid-transfer protein/seed storage 2S albumin superfamily protein; | Transport | 0.0076 | 1.00 | 2.587 |
| AT3G21500 | 1-deoxy-D-xylulose 5-phosphate synthase 1 (DXPS1) | 1-deoxy-D-xylulose 5-phosphate synthase 1; | Secondary metabolite synthesis | 0.0076 | 1.00 | 6.116 |
| AT1G77760 | nitrate reductase 1 (NIA1) | nitrate reductase 1; | Transport | 0.0076 | 1.00 | 0.564 |
| AT1G56610 | Protein with RNI-like/FBD-like domains | Protein with RNI-like/FBD-like domains; | N/A | 0.0076 | 1.00 | 0.584 |
| AT4G36791 | unknown protein | | N/A | 0.0077 | 1.00 | 100181140.400 |
| AT3G49750 | receptor like protein 44 (RLP44) | receptor like protein 44; | Signaling transduction | 0.0077 | 1.00 | 0.338 |
| AT4G12480 | pEARLI 1 | Bifunctional inhibitor/lipid-transfer protein/seed storage 2S albumin superfamily protein; | Transport | 0.0078 | 1.00 | 3.849 |
| AT5G08320 | CONTAINS InterPro DOMAIN/s: E2F-associated phosphoprotein, C-terminal (InterPro:IPR019370) | | N/A | 0.0078 | 1.00 | 2.769 |
| AT5G12940 | Leucine-rich repeat (LRR) family protein | Leucine-rich repeat (LRR) family protein; | Signaling transduction | 0.0078 | 1.00 | 0.518 |

| | | | | | | |
|---|---|---|---|---|---|---|
| AT5G09980 | elicitor peptide 4 precursor (PROPEP4) | elicitor peptide 4 precursor; | N/A | 0.0080 | 1.00 | 5.359 |
| AT3G54990 | SCHLAFMUTZE (SMZ) | Integrase-type DNA-binding superfamily protein; | Transcription factor | 0.0082 | 1.00 | 2.391 |
| AT5G23350 | GRAM domain-containing protein / ABA-responsive protein-related | GRAM domain-containing protein / ABA-responsive protein-related; | Transcription factor | 0.0082 | 1.00 | 6.301 |
| AT1G26390 | FAD-binding Berberine family protein | FAD-binding Berberine family protein; | Oxidation-reduction | 0.0083 | 1.00 | 6.282 |
| AT4G34230 | cinnamyl alcohol dehydrogenase 5 (CAD5) | cinnamyl alcohol dehydrogenase 5; | Secondary metabolite synthesis | 0.0084 | 1.00 | 1.970 |
| AT1G53165 | ATMAP4K ALPHA1 | Protein kinase superfamily protein; | N/A | 0.0085 | 1.00 | 0.588 |
| AT5G13500 | unknown protein | | N/A | 0.0085 | 1.00 | 1.634 |
| AT1G22570 | Major facilitator superfamily protein | Major facilitator superfamily protein; | Transport | 0.0086 | 1.00 | 3.367 |
| AT1G72850 | Disease resistance protein (TIR-NBS class) | Disease resistance protein (TIR-NBS class); | Defense | 0.0087 | 1.00 | 0.260 |
| AT3G22425 | imidazoleglycerol-phosphate dehydratase (IGPD) | imidazoleglycerol-phosphate dehydratase; | Protein synthesis | 0.0087 | 1.00 | 0.621 |
| AT1G61065 | Protein of unknown function (DUF1218) | Protein of unknown function (DUF1218); | N/A | 0.0088 | 1.00 | 4.272 |
| AT5G54510 | DWARF IN LIGHT 1 (DFL1) | Auxin-responsive GH3 family protein; | Hormone (Auxin) | 0.0090 | 1.00 | 1.746 |
| AT3G57230 | AGAMOUS-like 16 (AGL16) | AGAMOUS-like 16; | Transcription factor | 0.0092 | 1.00 | 0.667 |
| AT2G41550 | Rho termination factor | Rho termination factor; | Transcription factor | 0.0093 | 1.00 | 3.495 |
| AT2G17620 | Cyclin B2 | Cyclin B2;1; | Protein modification | 0.0094 | 1.00 | 3.324 |
| AT5G51190 | Integrase-type DNA-binding superfamily protein | Integrase-type DNA-binding superfamily protein; | Transcription factor | 0.0094 | 1.00 | 0.426 |
| AT4G20880 | ethylene-responsive nuclear protein / ethylene-regulated nuclear protein (ERT2) | ethylene-responsive nuclear protein / ethylene-regulated nuclear protein (ERT2); | N/A | 0.0094 | 1.00 | 0.632 |
| AT5G55050 | GDSL-like Lipase/Acylhydrolase superfamily protein | defense response; | Catabolism | 0.0094 | 1.00 | 2.110 |
| AT3G26410 | methyltransferases | methyltransferases;nucleic acid binding; | N/A | 0.0094 | 1.00 | 2.332 |
| AT1G74870 | RING/U-box superfamily protein | RING/U-box superfamily protein; | N/A | 0.0095 | 1.00 | 0.000 |
| AT3G07340 | basic helix-loop-helix (bHLH) DNA-binding | basic helix-loop-helix (bHLH) DNA-binding superfamily protein; | Transcription factor | 0.0096 | 1.00 | 1.844 |

| | | | | | | |
|---|---|---|---|---|---|---|
| | superfamily protein | | | | | |
| AT1G75120 | REDUCED RESIDUAL ARABINOSE 1 (RRA1) | Nucleotide-diphospho-sugar transferase family protein; | Sugar synthesis | 0.0096 | 1.00 | 2.940 |
| AT3G29810 | COBRA-like protein 2 precursor (COBL2) | COBRA-like protein 2 precursor; | N/A | 0.0096 | 1.00 | 2.926 |
| AT1G76060 | EMBRYO DEFECTIVE 1793 (EMB1793) | LYR family of Fe/S cluster biogenesis protein; | N/A | 0.0097 | 1.00 | 2.053 |
| AT1G16830 | Pentatricopeptide repeat (PPR) superfamily protein | Pentatricopeptide repeat (PPR) superfamily protein; | N/A | 0.0097 | 1.00 | 6.302 |
| AT2G42750 | DNAJ heat shock N-terminal domain-containing protein | DNAJ heat shock N-terminal domain-containing protein; | Protein modification | 0.0097 | 1.00 | 0.625 |
| AT2G39180 | CRINKLY4 related 2 (CCR2) | CRINKLY4 related 2; | Protein modification | 0.0097 | 1.00 | 3.223 |
| AT2G34060 | Peroxidase superfamily protein | Peroxidase superfamily protein; | Oxidation-reduction | 0.0098 | 1.00 | 0.410 |
| AT2G03010 | Family of unknown function (DUF577) | Family of unknown function (DUF577); | N/A | 0.0099 | 1.00 | 5.822 |
| AT1G28600 | GDSL-like Lipase/Acylhydrolase superfamily protein | GDSL-like Lipase/Acylhydrolase superfamily protein; | N/A | 0.0099 | 1.00 | 1.480 |
| AT5G15170 | tyrosyl-DNA phosphodiesterase-related | tyrosyl-DNA phosphodiesterase-related; | Nucleic acid modification | 0.0099 | 1.00 | 0.547 |
| AT4G23890 | unknown protein | | N/A | 0.0100 | 1.00 | 0.727 |

## References


Hutchinson, E.G., and Thornton, J.M. (1996). PROMOTIF—A program to identify and analyze structural motifs in proteins. *Protein Science* 5, 212-220. doi: 10.1002/pro.5560050204

Kozlowski, L.P., and Bujnicki, J.M. (2012). MetaDisorder: a meta-server for the prediction of intrinsic disorder in proteins. *BMC bioinformatics* 13, 111. doi: 10.1186/1471-2105-13-111

Kyte, J., and Doolittle, R.F. (1982). A simple method for displaying the hydropathic character of a protein. *J. Mol. Biol.* 157, 105-132. doi: 10.1016/0022-2836(82)90515-0

Li, L., Foster, C.M., Gan, Q., Nettleton, D., James, M.G., Myers, A.M., and Wurtele, E.S. (2009). Identification of the novel protein QQS as a component of the starch

metabolic network in Arabidopsis leaves. *Plant J.* 58, 485-498. doi: 10.1111/j.1365-313X.2009.03793.x

Li, L., Ilarslan, H., James, M.G., Myers, A.M., and Wurtele, E.S. (2007). Genome wide co-expression among the starch debranching enzyme genes AtISA1, AtISA2, and AtISA3 in Arabidopsis thaliana. *J. Exp. Bot.* 58, 3323-3342. doi: 10.1093/jxb/erm180

Mentzen, W.I., and Wurtele, E.S. (2008). Regulon organization of Arabidopsis. *BMC Plant Biol.* 8, 99. doi: 10.1186/1471-2229-8-99

O'connor, T.R., Dyreson, C., and Wyrick, J.J. (2005). Athena: a resource for rapid visualization and systematic analysis of Arabidopsis promoter sequences. *Bioinformatics* 21, 4411-4413. doi: 10.1093/bioinformatics/bti714

Sucaet, Y., Wang, Y., Li, J., and Wurtele, E.S. (2012). MetNet Online: a novel integrated resource for plant systems biology. *BMC bioinformatics* 13, 267. doi: 10.1186/1471-2105-13-267

Van Der Graaff, E., Schwacke, R., Schneider, A., Desimone, M., Flugge, U.I., and Kunze, R. (2006). Transcription analysis of arabidopsis membrane transporters and hormone pathways during developmental and induced leaf senescence. *Plant Physiol.* 141, 776-792. doi: 10.1104/pp.106.079293

Yamamoto, Y.Y., and Obokata, J. (2008). PPDB: a plant promoter database. *Nucleic Acids Res.* 36, D977-D981. doi: 10.1093/nar/gkm785
